# Preclinical evaluation of candidate “kill or cure” strategies to treat MFN2-related lipodystrophy

**DOI:** 10.1186/s10020-025-01314-2

**Published:** 2025-08-04

**Authors:** Ineke Luijten, Xiong Weng, Ula Kibildyte, Jana Buchan, Ami Onishi, Jake Mann, Eleanor McKay, David Savage, Robert K. Semple

**Affiliations:** 1 Centre for Cardiovascular Science, University of Edinburgh, Edinburgh, UK; 2 MRC Metabolic Diseases Unit, Institute of Metabolic Science, University of Cambridge, Cambridge, UK; 3 Department of Immunology and Immunotherapy, School of Infection, Inflammation and Immunology, College of Medicine and Health, University of Birmingham, Birmingham, UK; 4 Department of Biomedical Sciences, University of Lausanne, Lausanne, Switzerland; 5 MRC Human Genetics Unit, Institute of Genetics and Cancer, University of Edinburgh, Edinburgh, UK; 6 Centre for Cardiovascular Science, Queen’s Medical Research Institute, University of Edinburgh, Little France Crescent, Edinburgh, EH16 4TJ UK

**Keywords:** Mitofusin, MFN2, Lipodystrophy, Multiple symmetrical lipomatosis, Alcohol, Rapamycin, Sirolimus

## Abstract

**Background:**

The mitofusin 2 (MFN2) R707W mutation causes debilitating human lipodystrophy featuring lower body adipose loss, upper body adipose hyperplasia, and dyslipidaemic insulin resistance. Mechanical complications include airway compromise due to head and neck adipose overgrowth. This condition, sometimes called Multiple Symmetrical Lipomatosis (MSL), is also seen in sporadic form strongly associated with excess ethanol consumption. Mitigating the cellular pathology, or, conversely, exacerbating it, inducing selective death of affected adipocytes, are potential therapeutic strategies.

**Methods:**

Candidate exacerbating and mitigating approaches to MFN2-MSL were tested in human *MFN2*^*R707W/R707W*^ fibroblasts, and in *Mfn2*^*R707W/R707W*^ mice and derived preadipocytes. Cell survival, mitochondrial network morphology and integrated stress response markers were assessed in cells, and body composition and metabolic indices in mice.

**Results:**

Forcing galactose metabolism in human *MFN2*^*R707W/R707W*^ dermal fibroblasts did not replicate the overt adipose mitochondrial phenotype. 50mmol ethanol had little effect on *Mfn2*^*R707W/R707W*^ white preadipocytes, but increased mitochondrial content and blunted mitolysosome formation in *Mfn2*^*R707W/R707W*^ brown preadipocytes. 20% EtOH consumption increased brown adipose tissue in female *Mfn2*^*R707W/R707W*^ *mice*, and serum lactate in males. Rapamycin – a candidate mitigating treatment - increased size and mitolysosome content of WT preadipocytes, and to a lesser degree of *Mfn2*^*R707W/R707W*^ preadipocytes. In male *Mfn2*^*R707W/R707W*^ mice, rapamycin reduced weight gain, brown adipose mass, and increased serum Fgf21. Finally, a panel of mitochondrial stressors solicited no selective death or ISR in *Mfn2*^*R707W/R707W*^ preadipocytes.

**Conclusions:**

Ethanol mildly exacerbates murine *MFN2*-related MSL, while rapamycin is tolerated. MFN2-related MSL may not be solely attributable to compromised oxidative phosphorylation.

**Supplementary Information:**

The online version contains supplementary material available at 10.1186/s10020-025-01314-2.

## Background

We and others have described a severe and unusual form of lipodystrophy – pathological adipose tissue redistribution - caused by biallelic mutation of MFN2, encoding mitofusin 2 (Rocha 2017, Sawyer 2015, Capel 2018, Carr 2015, Pareyson 2015). Mitofusin 2 is an outer mitochondrial membrane protein best known to play a key role in mitochondrial fusion. It also mediates tethering of mitochondria to other organelles and structures including the endoplasmic reticulum and lipid droplets (Larrea 2019), some of this tethering mediated by shorter MFN2 isoforms (Naon 2023). Such apposition plays an important role in processes such as apoptosis, and mitophagy.

Heterozygous MFN2 mutations most commonly cause inherited sensorimotor neuropathy (Chung 2006, Verhoeven 2006, Neusch 2007). Many causal mutations are nonsense, frameshift or essential splice site mutations, indicating that neuropathy is caused by MFN2 haploinsufficiency. MFN2-related lipodystrophy, in contrast, has only been associated with biallelic mutations, invariably including at least one R707W allele. The large majority of affected people reported have been homozygous for MFN2 R707W, although compound heterozygosity for R707W and a presumed functionally null allele is also described (Rocha 2017, Sawyer 2015, Capel 2018, Carr 2015, Pareyson 2015). The corresponding R > W missense variant is also seen in the shorter MFN2 variants recently demonstrated to mediate endoplasmic reticulum tethering (Naon 2023). Not all patients with MFN2-related lipodystrophy have peripheral neuropathy, suggesting that penetrance and/or expressivity of MFN2 R707W-related neuropathy may be lower than that of lipodystrophy.

A fascinating and unexplained feature of MFN2-related lipodystrophy is the striking tissue- and adipose depot-selectivity of the phenotype despite ubiquitous *MFN2 *expression (Rocha 2017, Sawyer 2015, Capel 2018, Carr 2015, Pareyson 2015). As in other partial lipodystrophies, visceral adipose tissue is grossly unaffected. Subcutaneous white adipose tissue is severely affected, in contrast, with a remarkable craniocaudal pattern. Lower body, gluteofemoral adipose tissue is lost, which is hypothesised but not proven to be due to loss of adipocytes. In sharp distinction, upper body white adipose tissue undergoes progressive hyperplasia and expansion (Rocha 2017, Sawyer 2015, Capel 2018, Carr 2015, Pareyson 2015). Expansion is sometimes sufficient to occlude the airway, leading in some reported cases to premature death. This striking anatomical pattern has often led MFN2 R707W-related lipodystrophy to be called “Multiple Symmetrical Lipomatosis (MSL)” although true, encapsulated lipomas are not present. A closely similar sporadic form of MSL is strongly associated with excess consumption of alcohol (EtOH) (Lemaitre 2021).

Allied to the severe anatomical abnormality, affected people also show marked metabolic derangement, including insulin resistance, dyslipidaemia and fatty liver. Mitochondrial dysfunction is manifest as elevated serum lactate concentration, abnormal mitochondrial ultrastructure, and strong transcriptomic signatures of mitochondrial dysfunction and activation of the integrated stress response (ISR) in affected adipose tissue (Rocha 2017).

The combination of “mechanical” complications of upper body adipose hyperplasia and metabolic features of adipose failure imposes major morbidity. Metabolic complications can be addressed - as in other forms of lipodystrophy - by treatments inducing negative energy balance and offloading adipose tissue, but anatomical complications frequently require adipose debulking surgery. No targeted therapies are currently available, but two strategies seem worthy of consideration. Mitigating the underlying cellular pathology to slow or abrogate adipose remodelling is most appealing. One example of such an approach would be repurposing of mTOR inhibitors such as rapamycin (sirolimus), a strategy proposed for several different mitochondrial disorders based on model organism studies (e.g. (Johnson 2013, Khan 2017, Civiletto 2018, Siegmund 2017, Fan 2019)). An alternative novel approach could exploit synthetic lethality. In other words, if a treatment could be identified that selectively induces death of hyperplastic adipose tissue, then the mechanical component of the disease may be abolished. This would be at the expense of worsening deficiency of adipose tissue, but an established and growing suite of therapeutic approaches exist even for generalised adipose loss (Lim 2021). In effect, this strategy would resolve two major problems - metabolic and mechanical – into a metabolic problem alone.

To interrogate the molecular pathology of MFN2-linked lipodystrophy and facilitate preclinical translational studies we made, and have reported, *Mfn2*^*R707W/R707W*^mice (Mann 2023). White and brown adipose tissue (WAT and BAT respectively) from these mice recapitulated the microscopic adipose pathology described in humans, evidenced by consistent ultrastructural changes in mitochondrial morphology, transcriptomic evidence of activation of the ISR, and relative suppression of serum leptin and adiponectin. However despite prolonged challenge with high fat diet, no frank anatomical lipodystrophy was observed, and neither systemic insulin resistance nor other evidence of adipose failure was seen, attenuating the value of these mice as a disease model (Mann 2023).

In this study we sought to investigate both “kill” and “cure” strategies in cells and animals to treat MFN2 R707W-related adipose dysfunction. A secondary aim was to test strategies to exacerbate the previously described phenotype of *Mfn2*^*R707W/R707W*^ mice to enhance their utility as a disease model.

## Research design and methods

### Human dermal fibroblast studies

Dermal fibroblasts were derived from skin biopsies from healthy volunteers and three people with biallelic MFN2 mutations (P1: R707W/R343del; P2 and P3: R707W/R707W) as previously described (Rocha 2017). Informed consent was obtained and confirmed in writing as part of studies approved by the UK National Research Ethics Committee (studies 18/EE/0068 and 12/EE/0405). Fibroblasts were cultured in Dulbecco’s Modified Eagle’s Medium (DMEM) (Gibco 41966-029) supplemented with 10% fetal bovine serum (FBS) (Gibco 10500-064), 1% penicillin-streptomycin and 2 mM L-glutamine (Invitrogen), and 5 mM pyruvate, at 37 °C in 5% CO_2_/95% O_2_. For confocal studies, fibroblasts were grown on glass coverslips for 48 h in DMEM non-glucose medium containing 25 mM glucose (Glu) or 25 mM galactose (Gal) with 1% FBS, 1% penicillin-streptomycin and 2 mM L-glutamine (Invitrogen), and 5 mM pyruvate. Cells were labeled with MitoTracker (green, Molecular Probes) and Hoechst (blue) and then fixed with 4% paraformaldehyde for 15 min at room temperature. Coverslips were mounted in ProLong Gold Antifade Reagent. Imaging and analysis were undertaken as described for MitoQC-expressing cells.

### Mouse embryonic fibroblast (MEF) culture and treatment

MEFs were derived from *Mfn2*^*WT/WT*^, *Mfn2*^*WT/R707W*^ and *Mfn2*^*R707W/R707W*^embryos and immortalized with SV40 large T antigen as described previously (Mann 2023). All MEFs were grown at 37°C with 5% CO_2_ in DMEM containing 10% fetal bovine serum, Penicillin-Streptomycin-Glutamine (Gibco 10378-016), 1mM sodium pyruvate (Gibco 11360-070), MEM non-essential amino acid solution (Gibco 11140-050) and 50mM beta-mercaptoethanol (Gibco 31350-010).

To assess the effect of alcohol (EtOH) on MEF proliferation and viability, MEFs were seeded in triplicate at 2.5*10^4^ cells/ml (c.80% confluence), grown for 24 h, and then treated with the range of concentrations indicated of EtOH (absolute EtOH in H_2_O). After 24 h of treatment, cell number was determined first by MTT (3-(4,5-dimethylthiazolyl-2)−2,5-diphenyltetrazolium bromide, Sigma, TOX1-1KT) assay according to the manufacturer’s instructions (Abcam ab211091). All EtOH experiments with MTT assay were repeated 6 times.

Cell number and viability after 24 h EtOH treatment were also determined by Click-iT™ EdU Pacific Blue™ assay (ThermoFisher C10636). Growth medium was replaced with medium containing EtOH at concentrations indicated as well as 1mM EdU. After 2 h incubation, cells were detached, counted and 1*10^6^ cells resuspended in 100 ml Phosphate-buffered saline (PBS) containing 1% bovine-serum albumin and 400nM Apotracker™ Green (Biolegend 427402). Cells were incubated for 15 min at room temperature in the dark, before washing with PBS and resuspended in 100 ml PBS containing 1000x diluted reconstituted LIVE/DEAD™ fixable far red dead cell stain (ThermoFisher L34973). Cells were incubated for 30 min at room temperature in the dark, washed, and fixed in 4% paraformaldehyde (PFA). Permeabilization and Click-iT EdU detection were undertaken according to manufacturer’s instructions. Proliferation and apoptosis were determined using an Attune Nxt flow cytometer (ThermoFisher). The experiment was repeated 3 times.

### mRNA expression assays

For determination of gene expression, MEFs were washed with ice-cold PBS, harvested in TRIzol™ Reagent (ThermoFisher 15596018), and RNA was extracted according to the manufacturer’s instructions. cDNA was prepared from 500ng total RNA using the High-Capacity cDNA Reverse Transcription kit (ThermoFisher 4374966). Gene expression was measured in triplicate using the primers shown (Supplementary Table 1) and SYBR green dye (SYBR^®^ Green JumpStart™ *Taq* ReadyMix™, Sigma S5193) in a LightCycler^®^ 480 Instrument II (Roche). Specificity and efficiency of all primers was confirmed by standard dilution curve and agarose gel electrophoresis of PCR products prior to experimental reactions. Relative mRNA levels were determined using the ΔC_t_ method (2^−ΔCt^) using TATA-box binding protein (Tbp) as an endogenous control.

### Animal models and husbandry

All experiments were performed under UK Home Office Project License P87539BCC, approved by the University of Edinburgh Animal Welfare and Ethical Review Board, and conducted in line with ARRIVE guidelines. Generation of mice carrying the *Mfn2 *R707W mutation has been described previously (Mann 2023). *Mfn2*^*R707W/R707W*^ mice and WT littermate controls were derived by crossing *Mfn2*^*WT/R707W*^ mice on a C57Bl6/J background. Animals were group-housed in individually ventilated cages at 21°C with a 12 h/12 h light/dark cycle, controlled standard humidity (50%), and *ad libitum* access to water and chow diet (CRM, Special Diets Services). All genotyping was performed by Transnetyx (Cordova, TN). In tissue-based studies, no samples were excluded based on outcome or group identity – i.e. all available data that passed quality control were included in the final analysis.

To aid visualization of mitochondrial network structure and mitophagy, *Mfn2*^*WT/R707W*^mice were crossed with mice heterozygous or homozygous for the MitoQC reporter, described previously (McWilliams 2016). MitoQC mice were imported from MRC Harwell and housed under standard housing conditions as described above (Rhodes 2005).

### Mouse ethanol treatment

At 6 weeks of age, male and female *Mfn2*^*WT/WT*^ and *Mfn2*^*R707W/R707W*^mice were moved to a room with reversed light/dark cycle (dark 7AM-7PM, light 7PM-7AM). After 4 weeks’ acclimatisation, mice were single caged and weekly body weight, food intake (chow diet) and water intake measurements were made. At 12 weeks of age, body composition was measured by TD-NMR (Bruker Minispec Live Mice Analyzer LF50) and mice started on a 3-month Drinking-In-the-Dark (DID) regimen (Rath 2021), wherein water bottles were changed to bottles containing 20% (v/v) EtOH or vehicle (H_2_O) every day from 10-12AM. The DID regimen is an established model of voluntary binge-like ethanol consumption in rodents (Rath 2021, Thiele and Navarro 2014), and reliably induces high levels of ethanol intake over a short period. This mimics human binge drinking behaviour but without the confounding effects of chronic adaptation. Body composition was measured monthly before the start of daily EtOH drinking. Food consumption was monitored weekly, and bodyweight every 3 days. At the end of the study, body composition was measured and a terminal bleed undertaken by cardiac puncture under general anesthesia. Tissues were harvested immediately postmortem, weighed, and snap frozen.

### Mouse rapamycin treatment

At 7 weeks of age, male and female *Mfn2*^*WT/WT*^ and *Mfn2*^*R707W/R707W*^ mice were single caged at 21°C and provided with *ad libitum* access to a 45% HFD (Research Diets D12541). Body weight and food intake were measured weekly throughout the experiment. At 10 weeks of age, body composition was measured by TD-NMR (Bruker Minispec Live Mice Analyzer LF50) and mice were randomly assigned to receive intraperitoneal injections with vehicle (1%DMSO, 5% PEG400, 5% Tween-80) or 8 mg/kg rapamycin (VWR J62473) on alternate days for a month. After 4 weeks (at 14 weeks old), body composition was measured, and a terminal bleed undertaken by cardiac puncture under general anesthesia. Tissues were harvested immediately post-mortem, weighed, and snap frozen.

### Determination of metabolic efficiency

Food intake in kJ/day was calculated by dividing weekly food intake in g by 7 and multiplying by the following stated energy density of the diets: Chow CRM 10.74 kJ/g, HFD 19.8 kJ/g. The change in body composition during the two-week study period was calculated as (fat mass end– fat mass start) + (lean mass end– lean mass start) and converted from g to kJ using 39 kJ/g and 5 kJ/g as energy densities for fat and lean mass respectively. Metabolic efficiency was calculated by dividing the difference in body composition (in kJ) by the food intake (in kJ) over the same period, multiplied by 100.

### Serum/plasma biochemical assays

Non-fasted terminal blood samples were collected at the end of rapamycin or EtOH treatment periods. Blood glucose was measured immediately using an AccuChek Performa Nano meter. For lactate measurements, 40ul blood was collected into a fluoride EDTA tube and centrifuged immediately before freezing of plasma at − 80 °C. Remaining blood was allowed to clot for 15 min at room temperature after which serum was isolated by centrifugation and stored at −20 °C until analysis. All biochemical assays specified in Supplementary Table 2 were undertaken by the Medical Research Council Metabolic Diseases Unit Mouse Biochemistry Laboratory, Cambridge.

### Mfn2^R707W/R707W^/MitoQC + primary preadipocyte cultures

Primary preadipocyte cultures were made from inguinal white (iWAT) and pooled interscapular, cervical and axillary BAT tissue depots from male *Mfn2*^*WT/WT*^ and *Mfn2*^*R707W/R707W*^ mice, all either homozygous or heterozygous for the MitoQC transgene. 4-6-week-old mice were sacrificed by CO_2_and cervical dislocation, and adipose tissue depots were dissected, minced, filtered, and digested in several steps as described previously (Petrovic 2008). The resulting stromovascular fractions were resuspended in growth medium (DMEM (Gibco 41966-029) containing 10% newborn calf serum, 1x Penicillin-Streptomycin-Glutamine (Gibco 10378-016), 4nM insulin (Merck 19278), 10mM HEPES (Sigma H0887), 2mM glutamine (Sigma G7513), and 25 mg/ml sodium ascorbate (Sigma A4034), plated on chambered coverslips (Ibidi 80806), and kept at 37°C with 5% CO_2_.

For FCCP treatment, cells were grown for 4 days during which growth medium was changed twice. On the 4th day, cells were treated with Carbonyl cyanide-p-trifluoromethoxyphenylhydrazone (FCCP) (Abcam 120081) at a final concentration of 20mM or vehicle (DMSO) for 8 h. For EtOH treatment, cells were grown for 3 days during which growth medium was changed once. On the 3rd day, medium was replaced with medium containing 50mM EtOH or vehicle (H_2_O), and cells were incubated for 24 h. For rapamycin treatment, cells were grown for 1 day after which medium was replaced with medium containing 200nM rapamycin (VWR J62473) or vehicle (DMSO). Cells were incubated for 3 days during which medium containing rapamycin or vehicle was replaced once.

### Confocal microscopy of MitoQC-expressing cells

At the end of treatment periods, primary cultures of *Mfn2*^*R707W/R707W*^*/MitoQC +*cells were rinsed twice in DPBS at 37 °C and fixed in 3.7% formaldehyde in 200mM HEPES (pH7) for 15 min. Cells were rinsed again and stained with 0.5 mg/ml Hoechst (ThermoFisher 62249) in DPBS for 10 min in the dark. After two more rinses, 4 drops of mounting medium (Ibidi 50001) were added per well, and the chambered cover slips were kept in the dark at 4 °C until imaging. Cells were imaged using a Leica TCS SP8 confocal microscope with a HC PL APO 63x CS2 lens. Red and green (MitoQC), and blue (Hoechst) Z-stacks of 5–10 slices each were obtained and run through Huygens essential deconvolution software. The deconvoluted images were analyzed in Fiji using the MitoQC counter (Montava-Garriga 2020) and MiNA (Valente 2017) macros. To analyze mitochondrial network structure, a maximum intensity z-projection of the green channel was made, and contrast was increased by 25%. Individual cells were selected and run through the MiNA macro using the default settings and the following ridge detection setup: High contrast = 0, low contrast = 40, Line width = 5, Min line length = 5. To analyze red only loci (i.e. mitophagy), a maximum intensity z-projection of the red, green and blue channel was made and brightness was increased 20% in the red and green channels. Individual cells were selected and run through the MitoQC macro using the following settings: Green channel = 2, Red channel = 1, Smoothing radius = 1, Ratio threshold = 1. Data for individual cells was compiled in Excel and around 200 cells (divided over tissues from 3 mice) were analyzed per genotype, tissue, and treatment.

### Cell death assay

To assess response to mitochondrial stressors, primary white preadipocytes were isolated from the inguinal fat pads of 6–8 weeks old *Mfn2*^*WT/WT*^ and *Mfn2*^*R707W/R707W*^ mice (not expressing mitoQC) and cultured as described above. To assess response to mitochondrial stressors, cells were seeded in 12 well plates at 3 × 10^5^cells/well. The next day medium was replaced with DMEM containing either no glucose (Thermo, 11966025), low glucose (5mM; Sigma, 50997) or galactose (10mM, 25mM; Sigma 3646739) and 1%FBS. Mitochondrial stressors (Doxycycline; Sigma 24390145, Actinonin; Sigma A6671 or mitoblock-6; Clinisciences 303215-67-0) were added to the final concentrations shown. After 48 h, cell death was measured using the ReadyProbes™ cell viability image kit (Blue and Green; Thermo R37609) according to manufacturer’s instruction. In brief, cells were stained by stable NucBlue^®^ (Hoechst) for nuclei and NucGreen^®^ Dead for dead cells at room temperature for 30 min. Images were acquired on an EVOS fluorescent microscope (Life Technologies AMF4300) and the proportion of dead cells expressed as a percentage of total cell number.

### Statistical analyses

Statistical analysis was undertaken in GraphPad Prism version 10.3.1. All tests used are indicated in figure legends, and included Student’s T test, and one way or two way ANOVA, adapted for repeated measures where appropriate. Tukey’s or Sídák’s multiple comparisons tests were used where indicated. In vivo sample sizes drew on our experience of studying mouse metabolic disease models generally, and prior studies using comparable endpoints in *Mfn2*^*R707W/R707W*^ mice. No formal a priori power calculations were undertaken, although groups of 5–8 are accepted to capture most meaningful variation in commonly measured metabolic and body composition endpoints. *Post hoc* power analysis was avoided given its underestimation of power in the case of null findings. Normal distribution was assumed for all data, as the small numbers required by best practice in animal research precluded formal testing for normality.

## Results

### Human dermal fibroblast studies

We have previously reported that dermal fibroblasts established from people with MFN2 R707W-related lipodystrophy do not show overt mitochondrial network disruption nor gene expression changes when cultured in standard medium containing 25 mM glucose (Rocha 2017). A recent study of dermal fibroblasts from patients with sensorimotor neuropathy due to heterozygous MFN2 loss-of-function mutations also found no overt mitochondrial network abnormalities nor mitochondrial dysfunction in similar conditions (Dang 2022). When the cells were exclusively provided with galactose rather than glucose, however, mitochondrial network fragmentation and mitochondrial dysfunction were seen (Dang 2022). This was rationalised by the failure of galactose metabolism to pyruvate to generate net ATP, rendering cells dependent on mitochondrial oxidative phosphorylation when metabolising only galactose (Aguer 2011).

**Fig. 1:**
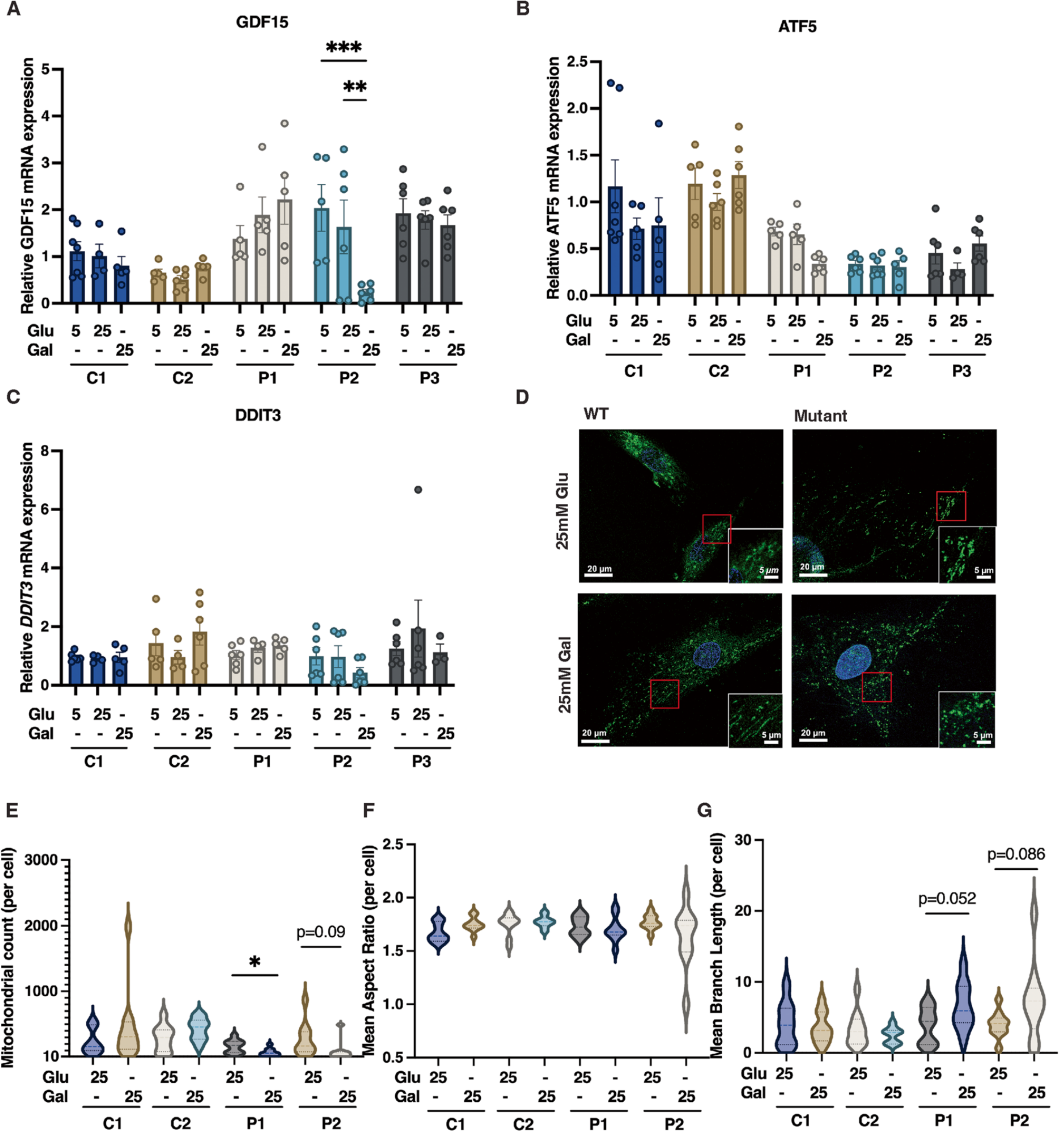
Effect of nutritional and pharmacological mitochondrial stressors on *MFN2*^*R707W/R707W*^ and wild-type (WT) dermal fibroblasts. **A-C**: Gene expression of GDF15, DDIT3, and ATF5 in human dermal fibroblasts from MFN2 WT healthy controls (C1,2), R707W homozygous (P1,2) or R707W/delR343 compound heterozygous (P3) patients cultured in medium containing 25 mM glucose (Glu), 25 mM galactose (Gal), or 5 mM glucose for 48 h. **D**: Representative microscopic images of mitochondria stained with MitoTracker (green) and Hoechst (blue) in human dermal fibroblasts cultured in medium containing 25 mM glucose or 25 mM galactose for 48 h. **E-G**: Quantification of mitochondrial counts, mean aspect ratio, and mean branch length, in the same experiment. Statistical analysis was undertaken using two-way ANOVA with Tukey’s multiple comparisons test. *p < 0.05, **p < 0.01, ***p < 0.001. *N* = 4 for gene expression and *N* = 10 per cell line and treatment for mitochondrial image analysis

Based on these findings we assessed whether forced reliance on galactose would also unmask mitochondrial dysfunction in *MFN2*^*R707W/R707W*^ dermal fibroblasts, thereby establishing a translationally tractable cellular disease model. We used mitochondrial morphology and transcriptional markers of the ISR as our primary readouts, based on observations of network fragmentation and a strong transcriptomic ISR signature in adipose tissue of both humans and mice homozygous for MFN2 R707W. Growth of fibroblasts from three patients in medium containing 25 mM galactose but no glucose for 48 h did not consistently increase expression of integrated stress genes *GDF15*, *DDIT3*, or *ATF5*, and indeed GDF15 expression fell in one patient (Fig. 1A-C). Confocal microscopy moreover revealed that although the mitochondrial network was fragmented in *MFN2*^*R707W/R707W*^ fibroblasts, this was seen whether cultured in 25 mM glucose or 25 mM galactose-containing medium, and no difference from wild-type (WT) control cells was observed in either condition (Fig. 1D). Galactose treatment did reduce the mitochondrial count per cell of *MFN2*^*R707W/R707W*^ but not WT cells (Fig. 1D, E). No differences in mean mitochondrial aspect ratio per cell were observed between genotypes or between treatments, and the mean mitochondrial branch length showed only a trend to an increase in *MFN2*^*R707W/R707W*^ - but not WT - cells on galactose treatment (Fig. 1D, F,G). We concluded that metabolic stress from galactose treatment was not sufficient to replicate the overt mitochondrial morphological and transcriptomic phenotype of affected adipose tissue in *MFN2*^*R707W/R707W*^ dermal fibroblasts.

### Evaluation of ethanol as a mitochondrial stressor in Mfn2^R707W/R707W^ cells

We next assessed a second mitochondrial stressor, namely ethanol (EtOH), for its ability to unmask or exacerbate cellular and physiological dysfunction associated with the Mfn2 R707W mutation. The choice of EtOH was based first on the well-established deleterious effect of EtOH on mitochondrial function, mediated in part by increased redox stress and mitochondrial DNA damage (Hoek et al. 2002). A second important motivation was the observation that excess EtOH consumption is strongly associated with the sporadic form of MSL, or Madelung’s disease, that closely resembles MFN2-related lipodystrophy (Lemaitre 2021).

To enable assessment of both cellular and physiological consequences of EtOH treatment we turned to the *Mfn2*^*R707W/R707W*^mice (Mann 2023), and murine embryonic fibroblasts (MEFs) derived from them. Initially, we assessed the effects of different concentrations of EtOH (0-100 mM) on viability of WT and homozygous MEFs. For reference, legal limits for blood alcohol when driving fall in the 10–18 mmol/L range in most countries. EtOH slightly increased the viability of homozygous MEFs, but did not affect WT cells (Supplementary Fig. 1A). No significant genotype- nor dose-dependent differences in cell proliferation or apoptosis were observed between WT and homozygous MEFs (Supplementary Fig. 1B, C). Determination of expression of a panel of genes related to mitochondrial biosynthesis and the ISR after 48 h of EtOH treatment revealed only an increase in *Pgc1b* expression in *Mfn2*^*R707W/WT*^ cells (Supplementary Fig. 1D), but this was present at baseline, was not exacerbated by EtOH treatment, and was not seen in homozygous cells. No EtOH-induced change in expression of *Pgc1α*, nor of genes involved in mitochondrial fusion and fission (*Mfn1*, *Mfn2*, *Drp1*, *Fis1*) was seen. More tellingly, no increase in expression of ISR genes (*Ddit3*, *Trib3*, *Atf4*, *Atf5*, *Gdf15*) was seen (Supplementary Fig. 1D), though they were sharply upregulated in adipose tissue from MFN2 R707W homozygous humans and mice (Rocha 2017, Mann 2023).

**Fig. 2:**
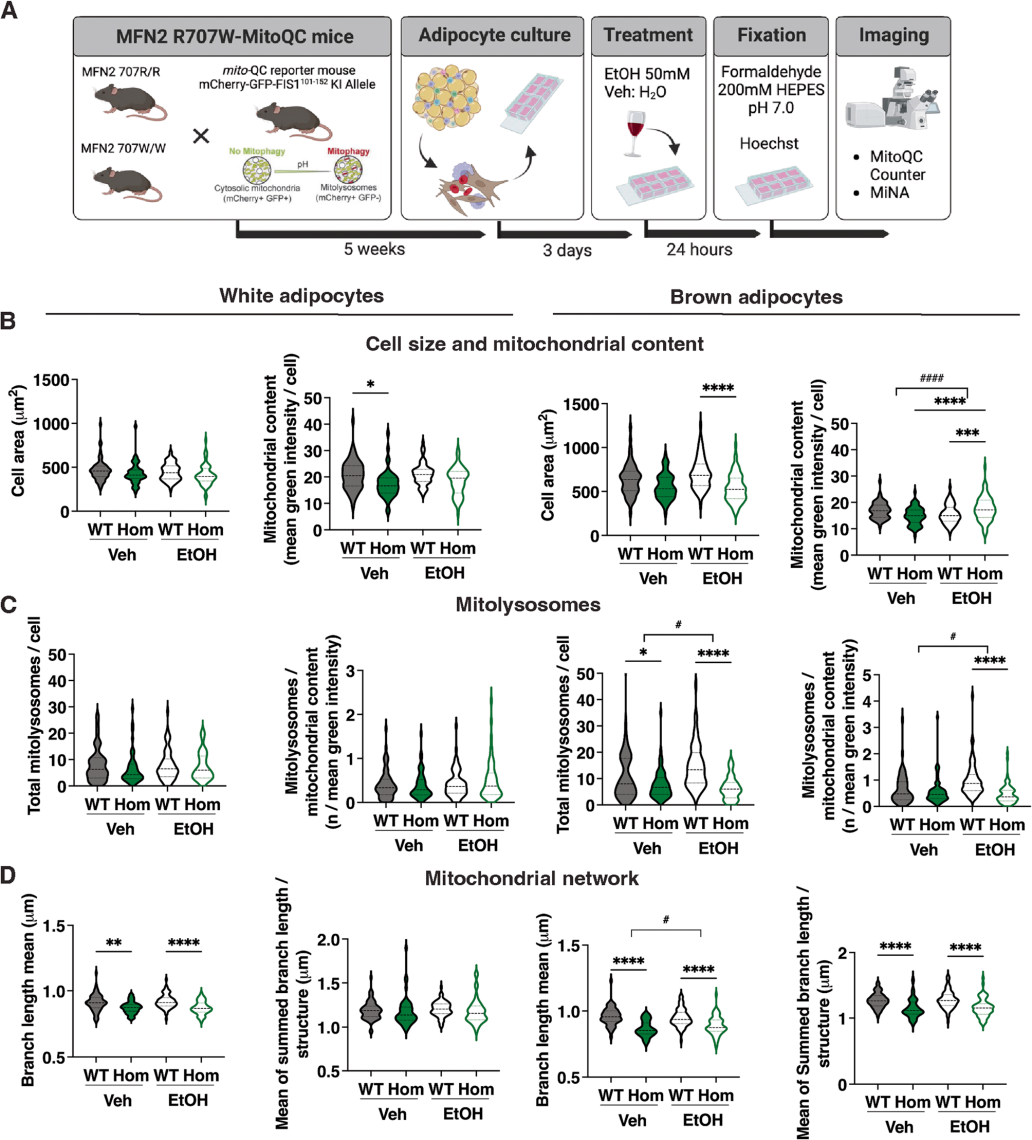
Effect of Ethanol (EtOH) on *Mfn2*^*R707W/R707W*^ (Hom) and wild-type (WT) primary preadipocytes. **A**: Schematic overview of the Mfn2 R707W x mito-QC breeding strategy, primary cell culture protocol, ethanol treatment, and mitochondrial imaging analysis (Created with *BioRender*). **B**: Cell area and mitochondrial content in primary white (left panels) or brown (right panels) adipocytes isolated from WT and Hom mice, cultured for 24 h in 50 mM EtOH or vehicle (Veh) **C**: Total mitolysosomes and mitolysosomes/mitochondrial content in experiment described in (B) **D**: Mean mitochondrial branch length and the mean of the summed mitochondrial branch length for each discrete mitochondrial structure in experiment described in (B). Statistical analysis was undertaken using two-way ANOVA with Tukey’s multiple comparisons test. *p < 0.05, **p < 0.01, ***p < 0.001, ****p < 0.0001; ^#^*p* < 0.05, ^####^*p* < 0.0001 for *Mfn2* genotype x treatment interaction

Our previous murine studies established that the cellular consequences of homozygosity for Mfn2 R707W are highly specific for adipose tissue, despite ubiquitous expression of Mfn2. Ultrastructural changes were discernible in preadipocytes in the earliest stages of lipid accumulation as well as in mature adipocytes (Rocha 2017). MEFs do have some limited adipose differentiation capacity but are not *bona fide* preadipocytes, and so may not be a valid disease model. We thus turned next to white and brown primary preadipocytes cultured from *Mfn2*^*R707W/R707W*^ mice. By using *Mfn2*^*R707W/R707W*^mice also transgenically expressing the MitoQC reporter (McWilliams 2016, Allen 2013), we were able to assess mitochondrial network morphology in cultured cells without using exogenous mitochondrial dyes. The MitoQC construct expresses a mitochondrially-targeted tandem mCherry-GFP. The mitochondrial network thus fluoresces red and green, but upon mitophagy, when pH falls in mitolysosomes, GFP fluorescence is selectively quenched, yielding mCherry-only foci as a readout of mitophagy (Fig.2A).

To test the model, we first assessed a raft of microscopic indices, including cell size, mitochondrial content, mitochondrial network morphology and mitolysosome number after exposure of primary mouse preadipocytes to carbonyl cyanide p-(trifluoromethoxy) phenylhydrazone (FCCP) (Supplementary Fig. 2A). FCCP is a lipid-soluble protonophore that collapses the mitochondrial proton gradient, inducing disintegration of the mitochondrial network, mitophagy and ultimately cell death. In white adipocytes, 8 h of treatment with 20µmol/L FCCP reduced mitochondrial content and mean branch length in the network, while greatly increasing the number of mitolysosomes, as expected (Supplementary Fig. 2B-E). In brown preadipocytes, FCCP again reduced network branch length, but had equivocal or no effect on mitochondrial content and mitolysosomes per cell, which were higher at baseline than in white preadipocytes (Supplementary Fig. 2F-I).

Having confirmed expected responses to FCCP in primary preadipocytes, we next tested the effect of 50mmol/L EtOH for 24 h (Fig. 2A). White preadipocytes showed only modest genotype-related changes, with reduced mitochondrial content in vehicle-treated cells only (Fig. 2B), and reduced mitochondrial branch length in both vehicle and EtOH-treated cells (Fig. 2D). EtOH exposure attenuated the difference in mitochondrial content seen in WT cells (Fig. 2B), but otherwise had no discernible morphological consequences. No change in mitolysosomes was seen across genotypes and EtOH treatment (Fig. 2C). Brown preadipocytes manifested greater morphological differences related both to genotype and to EtOH exposure. Specifically, there was a reduction in mean mitochondrial branch length and the mean sum of branch lengths in each discrete branching mitochondrial structure in *Mfn2*^*R707W/R707W*^ cells irrespective of EtOH exposure (Fig. 2D). Total and normalised mitolysosome content was lower in mutant than WT cells in both vehicle and EtOH-treated conditions, with the mutant appearing nearly abolishing the increases in both these indices induced by EtOH in WT cells. (Fig. 2C). EtOH had no significant effect on mitochondrial content in WT cells, but slightly increased this in *Mfn2*^*R707W/R707W*^ cells (Fig. 2B). In general, morphological differences between WT and *Mfn2*^*R707W/R707W*^ cells were exacerbated or unmasked in the presence of EtOH, with *Mfn2*^*R707W/R707W*^ cells showing lower cell area and increased mitochondrial content (Fig. 2B) and reduced mitolysosomes per cell (Fig. 2C) compared to WT cells only in the presence of EtOH.

### Evaluation of the effect of ethanol in vivo

**Fig. 3:**
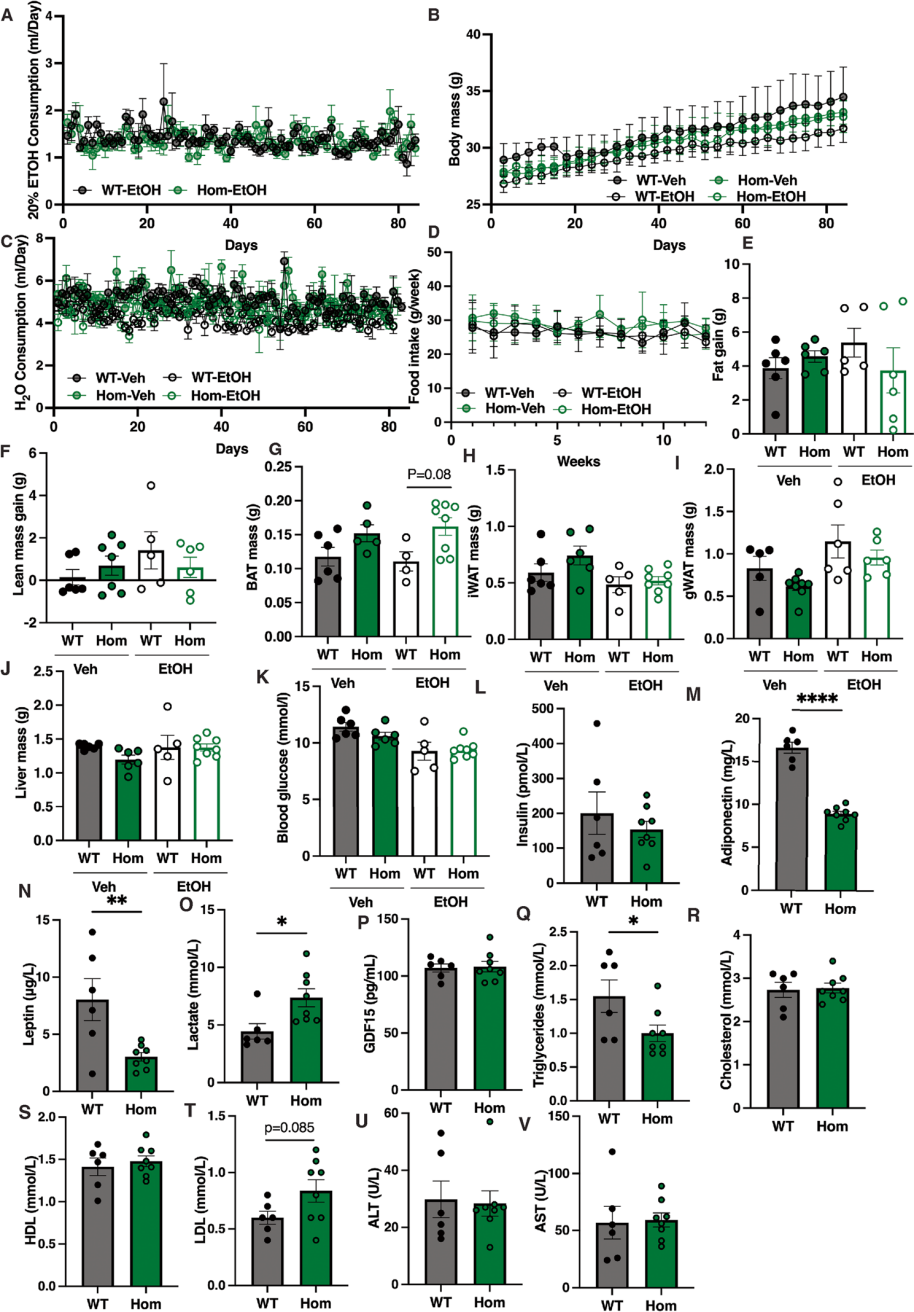
Effect of Ethanol (EtOH) on *Mfn2*^*R707W/R707W*^ (Hom) male mice and wild-type (WT) littermates. **A-D**: 20% EtOH consumption, Body weight, water consumption, and food intake in WT and Hom males during a 3 month “Drinking in the Dark” (DID) protocol with 20% ETOH or water control. **E-J**: Analysis of fat gain, lean mass gain, brown adipose tissue (BAT), inguinal (iWAT), gonadal white adipose tissue (gWAT), and liver mass in WT and Hom males at the end of the 3 months DID protocol. **K-V**: Serum levels of glucose, insulin, adiponectin, triglycerides, leptin, lactate, total cholesterol, HDL or LDL, ALT, AST, and Gdf15 in WT and Hom males at the end of the 3 months DID protocol. Statistical analysis was performed using Two-way ANOVA with Tukey’s multiple comparisons test or Student’s t-test. *p < 0.05, **p < 0.01, ***p < 0.001. *N* = 5–8 per group

Based on evidence from mouse primary cell studies that EtOH may exacerbate some aspects of the Mfn2 R707W-related cellular phenotype, we next evaluated the effect of 2 h per day of exposure to 20% EtOH in drinking water (Rath 2021) on WT and*Mfn2*^*R707W/R707W*^ mice over 12 weeks, starting from 12 weeks of age. WT and homozygous male mice consumed the same amount of 20% EtOH over the exposure period (Fig. 3A), but no genotype-related differences in body weight, food intake, nor overall water intake were seen (Fig. 3B-D). In keeping with this, no differences were seen in fat or lean mass gain (Fig. 3E, F). BAT mass trended towards an increase in homozygous mice both at baseline and after EtOH administration, with no observed differences in inguinal (iWAT), or gonadal (gWAT) WAT, nor liver mass (Fig. 3G-J).

Serum biochemical analysis was also undertaken at the end of EtOH exposure. This showed no differences in blood glucose or plasma insulin concentrations between genotypes (Fig. 3K, L). Serum concentrations of adipose tissue-derived adiponectin and leptin were significantly decreased in EtOH-treated homozygous mice (Fig. 3M, N), but only to a similar extent as in untreated mice in our prior studies (Mann 2023). Serum lactate, a common index of mitochondrial dysfunction, was increased in homozygous mice, unlike prior reports of animals on chow or high fat diet alone (Mann 2023), however serum Gdf15 concentration, a more general indicator of cellular stress (Lockhart et al. 2020), was not elevated (Fig.3O, P). Serum triglyceride concentration was reduced in EtOH-treated homozygous mice (Fig. 3Q), but no changes were seen in total, HDL or LDL cholesterol (Fig. 3R, T), nor in liver transaminases (Fig. 3U, V).

All in vivo studies of EtOH exposure were undertaken also in female mice (Supplementary Fig. 3). As in males, no divergence between WT and *Mfn2*^*R707W/R707W*^ mice was seen in EtOH consumption, body mass, food and water consumption, and change in fat and lean mass (Supplementary Fig. 3A-F). Also as in males, BAT mass was selectively increased (Supplementary Fig. 3G-J). Serum biochemical studies replicated the low serum leptin and adiponectin seen in males (Supplementary Fig. 3M, N), but lactate and triglyceride did not show any change (Supplementary Fig. 3O, Q), unlike in males. Other analytes were also unchanged (Supplementary Fig. 3K, L,P, R-V).

Collectively, our studies of the effects of exposure of *Mfn2*^*R707W/R707W*^ preadipocytes and animals to EtOH suggest that EtOH may exacerbate some cellular and organismal manifestations of Mfn2 R707W-related (pre)adipocyte dysfunction, especially in BAT, but the effect is mild. EtOH does not lead to a frank expression of the severe biochemical and anatomical phenotype seen in humans homozygous for MFN2 R707W.

### Assessment of the effect of Rapamycin on Mfn2^R707W/R707W^ cells

We next turned from environmental exposures that may worsen the MFN2 R707W lipodystrophy phenotype to assess a candidate treatment, namely the mTORC inhibitor rapamycin (sirolimus). Rapamycin is well known to upregulate mitochondrial biosynthesis, respiration, and mitophagy, thereby influencing cellular responses to nutrient and energy availability (Kennedy and Lamming 2016). It has also been suggested as a treatment option in several primary mitochondrial disorders (e.g. (Johnson 2013, Khan 2017, Civiletto 2018, Siegmund 2017, Fan 2019)). Furthermore, footprint analysis of bulk transcriptomic data from adipose tissue of humans with*MFN2*R707W-related lipodystrophy (Rocha 2017) and*Mfn2*^*R707W/R707W*^mice (Mann 2023) suggested that mTOR signalling was upregulated. Whether this was part of an adaptive response to mitigate the underlying genetic defect, or rather one of the mechanisms driving adipose hyperplasia, was not determined. This is a crucial translational question relevant to the safety of potential trials of rapamycin in people with MFN2 R707W-related lipodystrophy. Indeed, rapamycin has shown to be ineffective in some models of mitochondrial disorders (Barriocanal-Casado 2019), and to worsen clinically relevant outcomes in others (Ignatenko 2020).

**Fig. 4:**
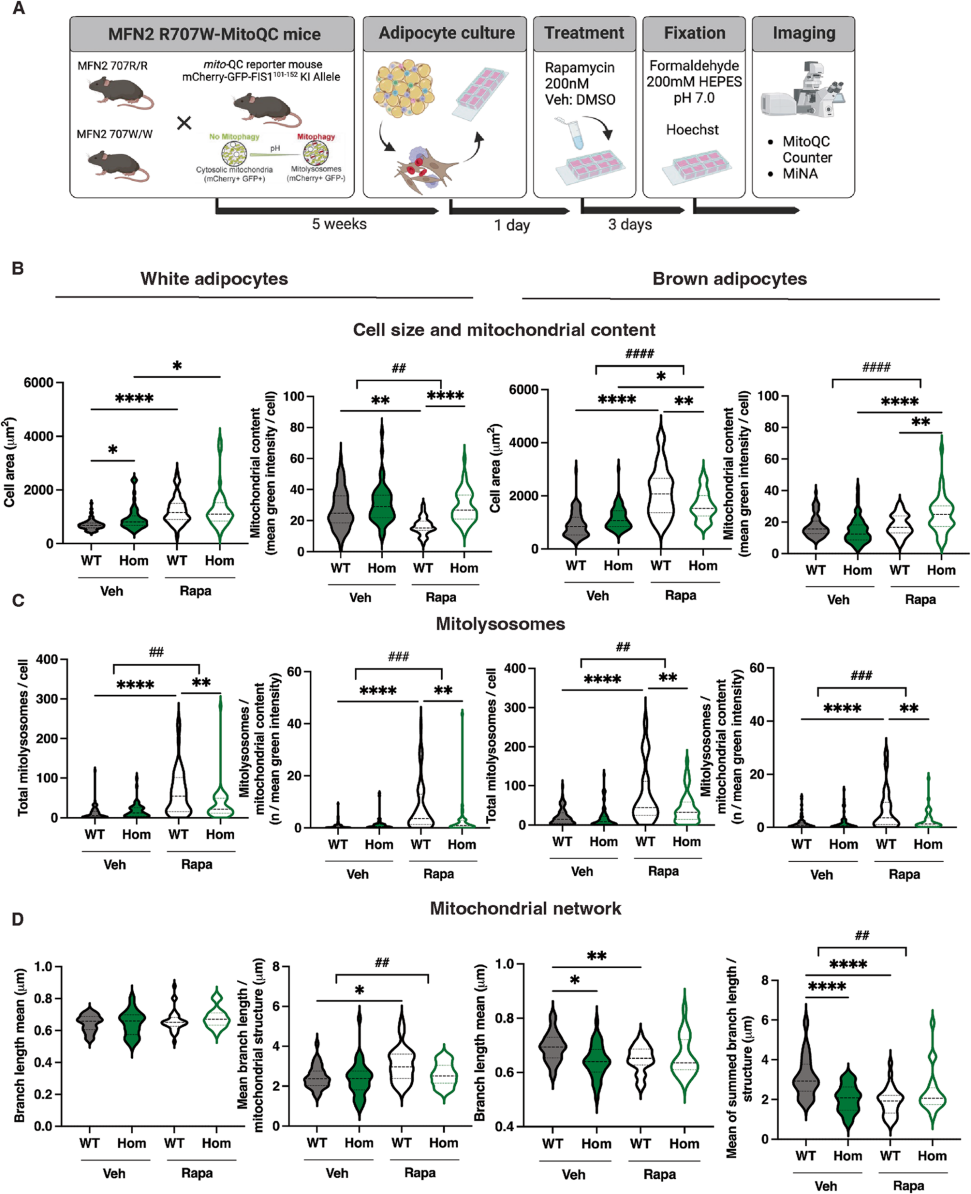
Effect of Rapamycin on *Mfn2*^*R707W/R707W*^ and wild-type (WT) primary preadipocytes. **A**: Schematic overview of the Mfn2 R707W x mito-QC breeding strategy, primary cell culture protocol, rapamycin treatment, and mitochondrial imaging analysis (Created with *BioRender*). **B**: Cell area and mitochondrial content in primary white (left panels) or brown (right panels) adipocytes isolated from WT and Hom mice, cultured for 72 h in 200nM rapamycin or vehicle (veh). **C**: Total mitolysosomes and mitolysosomes/mitochondrial content in experiment described in (B). **D**: Mean mitochondrial branch length and the mean of the summed mitochondrial branch length for each discrete mitochondrial structure in experiment described in (B). Statistical analysis was performed using Two-way ANOVA with Tukey’s multiple comparisons test. *p < 0.05, **p < 0.01, ***p < 0.001, ****p < 0.0001.; ^##^*p* < 0.01, ^###^*p* < 0.001, ^####^*p* < 0.0001 for *Mfn2* genotype x treatment interaction

Before undertaking in vivo studies, we first evaluated the effects of rapamycin on mitochondrial morphology and function in primary adipocytes, as before. Primary preadipocytes from WT and *Mfn2*^*R707W/R707W*^ mice, all co-expressing MitoQC, were isolated, and treated with 200nM rapamycin or vehicle for 72 h (Fig. 4A). Rapamycin treatment enlarged both white and brown adipocytes, but had variable effects on mitochondrial content across genotypes and cell types, with the clearest effect being an increase in *Mfn2*^*R707W/R707W*^ brown preadipocytes (Fig. 4B). In keeping with its known mitophagy-inducing effect, rapamycin sharply increased total and normalised mitolysosome numbers in WT cells, with significantly attenuated increases in *Mfn2*^*R707W/R707W*^ white and brown preadipocytes (Fig. 4C). Effects of rapamycin on mean mitochondrial branch length and the sum of branch lengths per discrete structure were variable and small, with the only consistent effect being reduction in branch length in both white and brown WT preadipocytes (Fig. 4D).

**Fig. 5:**
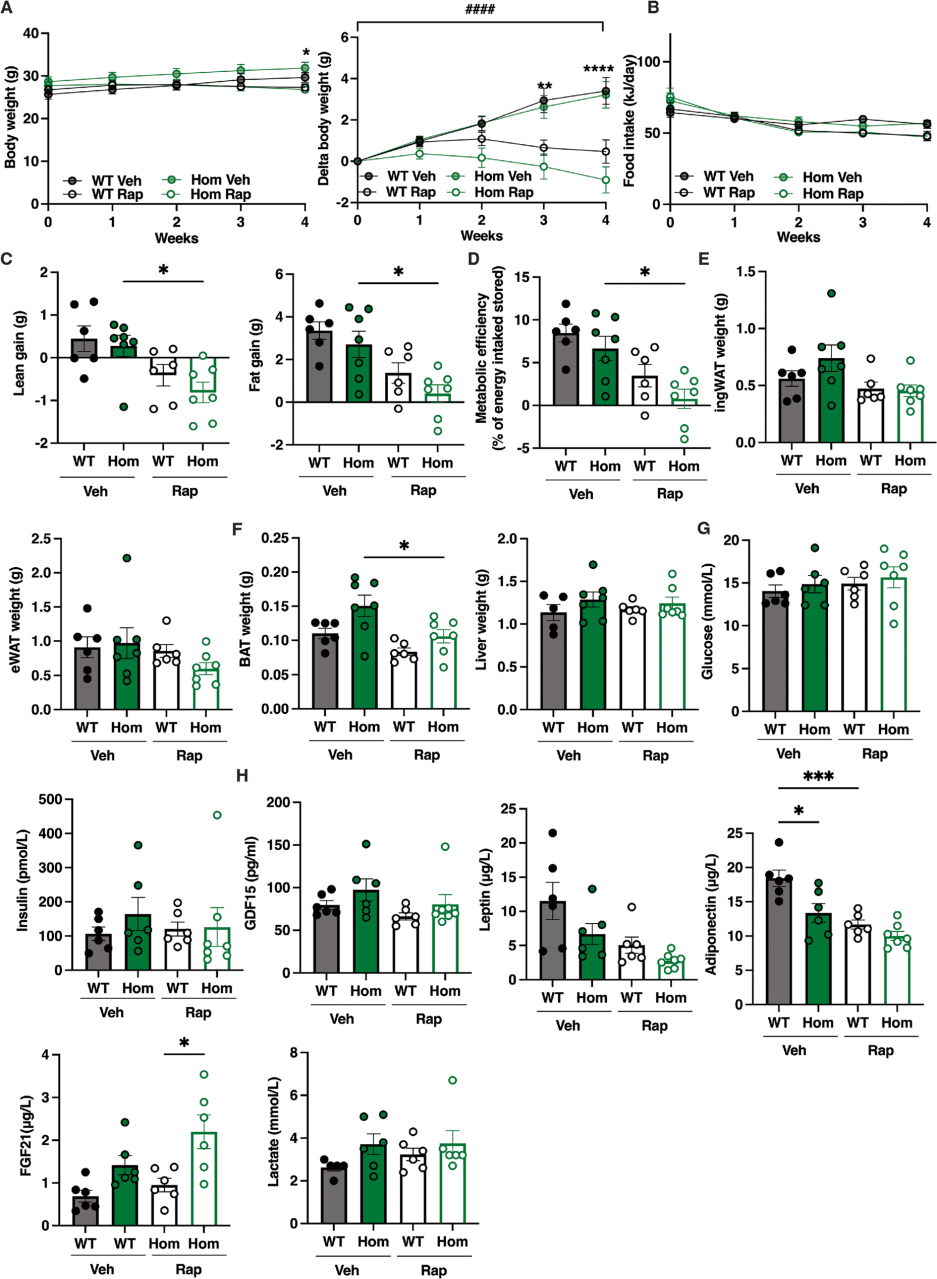
Effect of Rapamycin Treatment on male *Mfn2*^*R707W/R707W*^ mice and wild-type (WT) littermates.** A**: Body weight and body weight gain in male mice on a 45% HFD receiving intraperitoneal injections with 8 mg/kg rapamycin or vehicle (veh) every other day for 4 weeks. **B**: Food intake during rapamycin or veh treatment. Statistical analysis used repeated measures two-way ANOVA with Sídák’s multiple comparisons test; ^####^*p* < 0.0001 for *Mfn2* genotype x time x treatment interaction; ** = *p* < 0.01 and *** = *p* < 0.0001 for vehicle vs. rapamycin for both genotypes at the time points indicated **C**: Lean and fat mass gain over the 4-week treatment period. **D**: Metabolic efficiency calculated over the 4-week treatment period. **E**: iWAT and eWAT weight at the end of the 4-week treatment period. **F**: BAT and liver weight at the end of the 4-week treatment period. **G**,** H**: Plasma levels of glucose, insulin, GDF15, leptin, adiponectin, FGF21, and lactate at the end of the 4-week treatment period. Statistical analysis in (C-H) used Student’s T-test. * = *p* < 0.05. *N* = 6–7 per group

We next assessed the effects of rapamycin in vivo. High fat diet-fed mice were administered 8 g/kg Rapamycin or vehicle intraperitoneally on alternate days for four weeks, starting three weeks into 45% high-fat diet (HFD) feeding, which in turn was started at 7 weeks. Rapamycin markedly attenuated or reversed bodyweight gain without any effect on food intake in male mice (Fig. 5A, B). In *Mfn2*^*R707W/R707W*^ mice, rapamycin accentuated the loss of lean mass and decrease in fat mass gain seen in WT controls, and also increased the suppression of metabolic efficiency also seen in controls (Fig. 5C, D). No significant differences in inguinal (iWAT) and epididymal white adipose (eWAT) mass were seen in either genotype following rapamycin treatment, but BAT mass was reduced significantly in *Mfn2*^*R707W/R707W*^ mice only, while liver mass was unchanged (Fig. 5E, F). Rapamycin did not change plasma insulin or blood glucose in any group, but tended to reduce serum leptin and adiponectin, although this was only significant for adiponectin in WT mice. Neither lactate nor Gdf15 was altered by rapamycin in any group, but Fgf21 was increased in *Mfn2*^*R707W/R707W*^ mice (Fig. 5G, H).

The same study of rapamycin was undertaken in female mice, however almost no discernible effect of rapamycin was seen across the traits studied with the exception of a minor increase in liver weight in *Mfn2*^*R707W/R707W*^ mice only (Supplementary Fig. 4).

These findings do not suggest any obviously greater adverse effect on metabolism or body composition of rapamycin in *Mfn2*^*R707W/R707W*^ mice compared to WT controls. Effects of rapamycin were more pronounced in male than female mice, and these effects appeared to be accentuated in *Mfn2*^*R707W/R707W*^ mice.

### Assessment of further mitochondrial stressors on primary murine white preadipocytes

Having failed to observe severely deleterious or beneficial effects of EtOH and rapamycin respectively in *Mfn2*^*R707W/R707W*^ mice, we finally undertook a pilot study to assess other strategies to exacerbate the *Mfn2*^*R707W/R707W*^-related cellular phenotype, both with a view to improving fidelity of the murine model to the human condition, and to establishing a proof of principle for strategies to selectively kill overgrowing adipose tissue in affected people. For this pilot study we elected to concentrate on murine white preadipocytes cultured from inguinal fat pads, given their disease relevance and the tissue selectivity of the human and mouse *Mfn2*^*R707W/R707W*^ phenotype, using transcriptional hallmarks of the ISR as sensitive and convenient readouts of cellular pathology.

**Fig. 6:**
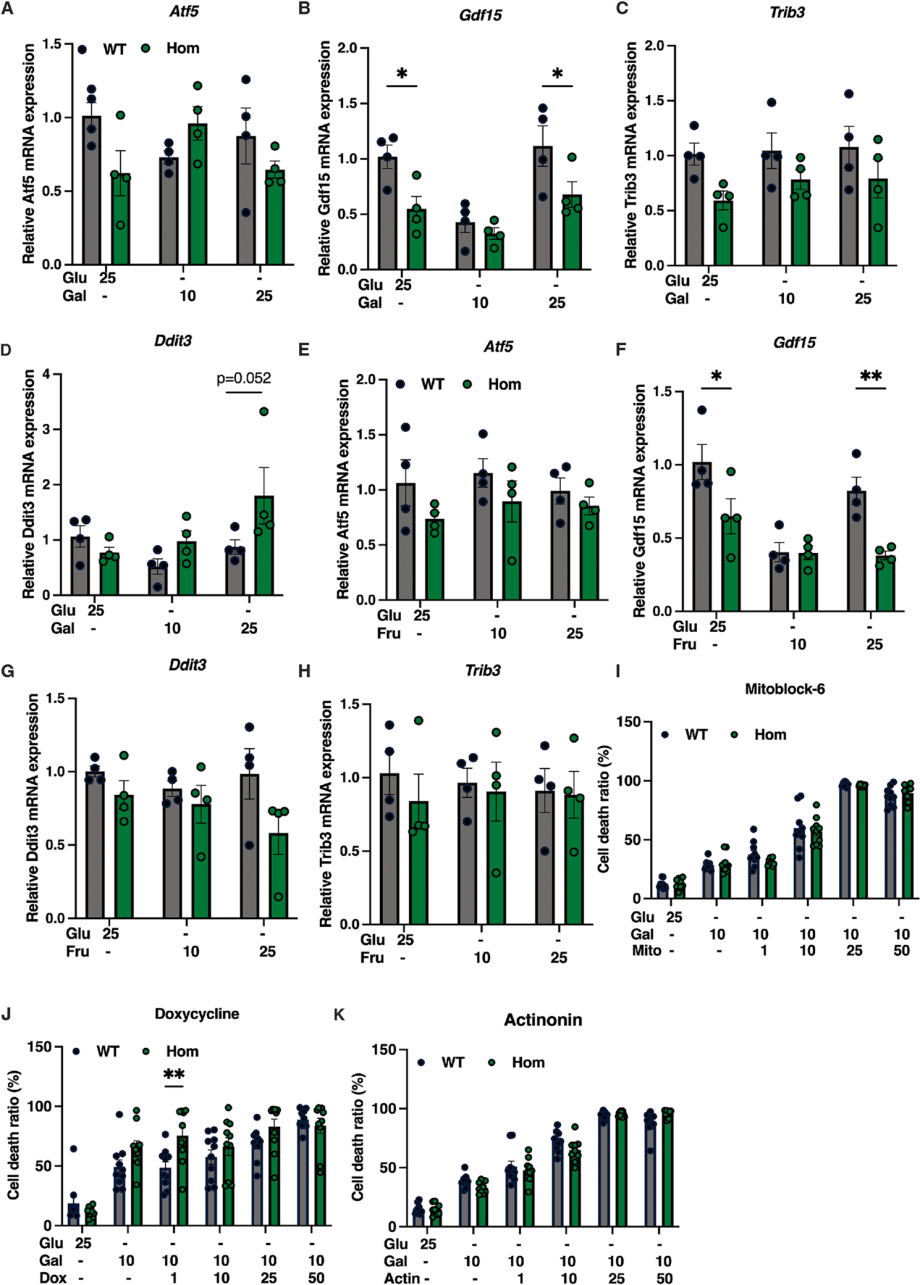
Effect of mitochondrial toxins on *Mfn2*^*R707W/R707W*^ and wild-type (WT) primary white preadipocytes. **A-D**: Gene expression of *Atf5*,* Gdf15*,* Trib3*,* and Ddit3* in mouse MFN2 WT and homozygous primary white preadipocytes cultured from inguinal fat pads and treated with 10 mM, 25 mM galactose, or 25 mM glucose as a control for 48 h. **E-H**: Gene expression of *Atf5*,* Gdf15*,* Trib3*,* and Ddit3* in mouse MFN2 WT and hom primary preadipocytes treated with 10 mM, 25 mM fructose (Fru), or 25 mM glucose as a control for 48 h. **I-K**: Quantification of cell death ratios in mouse MFN2 WT and homo primary preadipocytes treated with doxycycline, Mitoblock-6, and Actinonin (*N* = 6–10). Cells were treated with potential stressors for 48 h in DMEM without glucose, with 1% FBS, 2 mM glutamine, and 10 mM galactose or 20 mM glucose as a control. Cells were stained with stable NucBlue^®^ (Hoechst) for nuclei and NucGreen^®^ Dead for dead cells at room temperature for 30 min. Images were taken, and the dead cell ratio was calculated as the percentage of dead cells to total cell numbers. Statistical analysis used repeated measures two-way ANOVA with Sídák’s multiple comparisons test. *p < 0.05, **p < 0.01. *N* = 4 for gene expression and *N* = 10 for cell death assay

We first assessed the impact of nutritional stressors. The first of these was galactose, as in human fibroblast studies (Fig. 1). 48 h of growth in medium containing galactose but no glucose did not increase expression of *Atf5* and *Trib3* in cells of either genotype, and indeed *Gdf15* expression was reduced in *Mfn2*^*R707W/R707W*^ compared to WT cells in either glucose- or galactose-containing medium, while *Atf5* and *Trib3* trended lower in *Mfn2*^*R707W/R707W*^ cells. Only *Trib3* tended to increase in high galactose medium (Fig. 6A-D).

The second stressor tested was fructose. Forced fructose metabolism has been reported to impair mitochondrial function and activate the ISR in several tissue contexts, contributing to metabolic disturbances (Softic 2019).*SLC2A5*, encoding the GLUT5 fructose transporter, was moreover strongly upregulated in affected human adipose tissue in MFN R707W-related lipodystrophy (Rocha 2017). We used a similar experimental design as for galactose, replacing glucose in culture medium with low or high concentration fructose. Fructose did not increase expression of*Ddit3*, *Atf5*, or *Trib3* in either WT or *Mfn2*^*R707W/R707W*^ preadipocytes, and indeed, similarly to the prior experiment, *Gdf15* expression was reduced in *Mfn2*^*R707W/R707W*^ preadipocytes exposed to either 25 mM glucose or fructose (Fig. 6E-H).

We ended by testing three further small molecule mitochondrial stressors, using a previously reported rationale (Quiros 2017), and assessing them in the context of medium containing 10 mM galactose rather than glucose. In this final experiment we used cell death as the experimental readout as we were interested in potential synthetic lethality in the context of*Mfn2*R707W homozygosity. The first stressor assessed was Mitoblock-6, a small molecule that disrupts mitochondrial protein import by inhibiting Erv1/Mia40 and TIM13 (Dabir 2013). Mitoblock-6 did induce dose-dependent preadipocyte death, but no Mfn2 genotype-dependency of the effect was observed (Fig.6I). The second stressor examined was doxycycline, a common tetracycline that inhibits mitochondrial translation, thereby provoking mitochondrial integrated stress activation in many cell types (Moullan 2015). Doxycycline did not significantly increase cell death in a dose-dependent way, and no clear difference in response was discernible by genotype (Fig.6J). The final stressor tested was Actinonin, a small molecule that alters stability and synthesis of OXPHOS proteins (Richter 2015). Actinonin, like Mitoblock-6, provoked dose-dependent cell death, but again this effect did not show any genotype-dependency (Fig.6K). These findings do not support use of any of these approaches in translational strategies based on synthetic lethality.

## Discussion

MFN2 R707W, uniquely among the many pathogenic MFN2 variants described to date (Zuchner 1993), causes major morbidity and mortality due to a combination of adipose overgrowth and metabolic complications of lipodystrophy (Rocha 2017, Sawyer 2015, Capel 2018, Carr 2015, Pareyson 2015). There is a large unmet need to develop targeted therapy, ideally able to address both trophic and metabolic complications of the condition, but the lack of fidelity of cellular and in vivo disease models described to date is a barrier to translational research.

Given ubiquitous MFN2 expression, the cellular dysfunction caused by the R707W variant is remarkably selective, with only adipose tissue and to some extent peripheral nerves documented to be affected in humans (Rocha 2017, Sawyer 2015, Capel 2018, Carr 2015, Pareyson 2015). Primary dermal fibroblasts, although expressing MFN2 strongly, appear largely normal (Rocha 2017), while the mouse model we previously described does not phenocopy the adipose overgrowth and systemic metabolic derangements seen in humans. It does, however, replicate key aspects of the adipose pathology, including mitochondrial network fragmentation, activation of the ISR in adipose tissue, and suppressed adipokine secretion (Mann 2023).

One motivation for the current study was to assess whether additional stressors may unmask or increase cellular defects selectively in *Mfn2*^*R707W/R707W*^ cells and mice. One of these, EtOH, did show some evidence of a genotype-selective effect, increasing brown adipose tissue mass in female *Mfn2*^*R707W/R707W*^mice and a trend to an increase in males, and serum lactate in male mice only. EtOH also increased or unmasked mitochondrial morphology differences between WT and mutant brown preadipocytes, suggesting that EtOH does indeed exacerbate the murine phenotype, albeit not yet offering sufficient dynamic range in the phenotype induced to serve as a robust translational disease model. This effect of EtOH is interesting given the very strong association of excess EtOH consumption with the sporadic form of MSL (Lemaitre 2021). The current findings suggest that deranged MFN2 function may increase susceptibility to EtOH-induced MSL. Of note, we elected to use the established “Drinking in the Dark” experimental paradigm, which models human binge drinking through exposure to EtOH for 2 h each day (Rath 2021, Thiele and Navarro 2014). Whether other paradigms featuring more sustained EtOH exposure would solicit a greater effect on*Mfn2*^*R707W/R707W*^ mice is an interesting open question.

More surprisingly, given clear evidence of mitochondrial dysfunction in adipose tissue in MFN2 R707W homozygous humans and mice, no convincing mutant-selective sensitivity was seen for either cells and animals to other, more specific, exogenous mitochondrial stressors. Stressors tested were nutritional/metabolic (forced galactose or fructose metabolism in cells) and/or pharmacological (doxycycline, mitoblock-6 or actinonin in cells). This may be an indication that impaired oxidative phosphorylation (OxPhos) is not the mechanism mediating MFN2-related lipodystrophy, even though impaired OxPhos is seen in vivo (evidenced by increased serum lactate, and transcriptomic signatures in affected tissue (Rocha 2017). It also suggests that the relevant effect of EtOH that exacerbates the phenotype may not simply be to further impair OxPhos.

Mitochondria are often characterised simply as ATP-generating cellular “powerhouses”, but actually play a wide array of more nuanced roles. This reflects the need to adapt energy production and intermediary metabolism to different environmental conditions, and the needs of different organelles for fluxes of substrate and/or ions at different times for processes such as apoptosis and mitophagy. MFN2 subserves several functions relevant to these wider roles, mediating apposition of mitochondria to other organelles including the endoplasmic reticulum (de Brito and Scorrano 2008), enabling apoptosis and mitophagy, and lipid droplets, facilitating processes such as mitophagy and apoptosis (Rambold et al. 2015). It is plausible that the specific lipodystrophy-relevant function of MFN2 disturbed by the R707W mutation relates to tethering of mitochondria to other organelles rather than gross OxPhos dysfunction. It was recently demonstrated that two shorter splice variants of MFN2 may mediate ER tethering of mitochondria and ER morphology (Naon 2023), and, interestingly, the equivalent R > W missense mutation is retained in the heptad repeat domains in each of these shorter gene products also.

The contention that it may be perturbed mitochondrial-ER interactions that underlie MFN2-related lipodystrophy remains to be tested. However the wide range of different genetic disorders of mitochondrial function now known, which feature a broad range of severity of OxPhos dysfunction (Gorman 2016), give further credence to the notion that there is more to MFN2 R707W-associated lipodystrophy than simply impaired OxPhos. For despite the wider range of degrees of OxPhos impairment described, lipodystrophy is reported in an extremely small subset only of mitochondrial cytopathies. These include MERRF, caused in most cases by a mitochondrial mutation in the lysyl-tRNA gene (Larsson 1995), and some complex disorders caused by defects in nuclear-encoded mitochondrial genes (e.g. MTX2 (Elouej 2020) and TOMM7 (Garg 2022), both localised, like MFN2, to the mitochondrial outer membrane, and TYMP (Gautheron 2022), a cytosolic enzyme indirectly important in maintaining mitochondrial DNA). Whether there is mechanistic commonality underpinning these select mitochondrial disorders that cause lipodystrophy is unknown but may be a fruitful line of enquiry.

The second important question addressed by this study was whether inhibition of mTOR with rapamycin is likely to be safe in MFN2 R707W-related lipodystrophy. Rapamycin had the pronounced effects on cell biology and in vivo metabolism expected, but we failed to see any evidence of interaction between genotype and rapamycin in vivo or ex vivo. This suggests no obvious selective toxicity of rapamycin in *Mfn2*^*R707W/R707W*^ mice or cells, increasing confidence in the safety of any future trials.

## Conclusions

Ethanol mildly exacerbates murine Mfn2-related MSL, suggesting a gene environment interaction potentially relevant to sporadic, alcohol-related MSL. Rapamycin failed to exert genotype-specific clearly beneficial effects in cells and animals. On the other hand was well tolerated, alleviating concern that inhibiting secondary mTOR activation may cause decompensation and harm. This helps to derisk any potential human use in MFN2-MSL. The aims to either mitigate MFN2 R707W-related cellular dysfunction to arrest or reverse upper body adipose overgrowth (“cure”) in MSL, or instead to exploit synthetic lethality to selectively kill affected adipose tissue (“kill”) both remain valid. However, realising either of these strategies will require a wider, more agnostic screen of candidate interventions. As well as considering pharmacological approaches, future screens may also seek genetic modifiers of the phenotype, including knockdown of the Mfn2 paralogue Mfn1, which has been shown in some contexts to compensate for at least some neuropathy-causing mitofusin 2 mutations (Detmer and Chan 2007).

## Supplementary Information

Supplementary Material 1: Supplementary Figure 1: Effect of ethanol on wild-type and *Mfn2*^*R707W/R707W*^ mouse embryonic fibroblasts. A: Formazan formed in *Mfn2*^*WT/WT*^, *Mfn2*^*WT/R707W*^and *Mfn2*^*R707W/R707W*^MEFs treated with a range of EtOH concentrationsfor 24 h expressed as a percentage of vehicle treatment. B: EdU positive cells in MEFs as in, expressed as a percentage of live cells. C: Apotracker positive cells in MEFs as in, expressed as a percentage of live cells. D: Gene expression for* Pgc1a, Pgc1b, Mfn1, Mfn2, Drp1, Fis1, Ddit3, Trib3, Atf4, Atf5* and *Gdf15* in MEFs as in, normalized to reference gene *Tbp*. Statistical analysis was performed using Two-way ANOVA with Tukey’s multiple comparisons test. *p < 0.05, **p < 0.01. *N* = 6, Dotted vertical lines indicate legal limits for blood alcohol when driving. Supplementary Figure 2: Validation of the MitoQC Reporter using FCCP. A: Schematic overview of the Mfn2 R707W x mito-QC breeding strategy, primary cell culture protocol, FCCP treatment, and mitochondrial imaging analysis. B,F: Representative images of primary adipocytes isolated from iWATor BATof R707W x mitoQC mice, treated with 20uM FCCP or Veh for 8h. Green: mitochondrial network, red: mitolysosomes, blue: nuclei. C-E, G-I: mitochondrial content, total mitolysosome, and branch length quantifications of the experiment described in B. Statistical analysis was performed using Two-way ANOVA with Tukey’s multiple comparisons test. *p < 0.05, **p < 0.01, ***p < 0.001, ****p < 0.0001. Supplementary Figure 3: Effect of Ethanolon *Mfn2*^*R707W/R707W*^female mice and wild-typelittermates. A-D: 20% EtOH consumption, Body weight, water consumption, and food intake in WT and Hom males during a 3 month“Drinking in the Dark”protocol with 20% ETOH or water control. E-J: Analysis of fat gain, lean mass gain, brown adipose tissue, inguinal, gonadal white adipose tissue, and liver mass in WT and Hom males at the end of the 3 months DID protocol. K-V: Serum levels of glucose, insulin, adiponectin, triglycerides, leptin, lactate, total cholesterol, HDL or LDL, ALT, AST, and Gdf15 in WT and Hom males at the end of the 3 months DID protocol. Statistical analysis was performed using Two-way ANOVA with Tukey’s multiple comparisons test or Student's t-test. *p < 0.05, **p < 0.01, ***p < 0.001. *N* = 5-7 per group. Supplementary Figure 4: Effects of rapamycin treatment on Mfn2^R707W/R707W^ female mice and wild-type littermates. A: Body weight and body weight gain in female mice on a 45% HFD receiving intraperitoneal injections with 8mg/kg rapamycin or vehicleevery other day for 4 weeks. B: Food intake during rapamycin or vehicle treatment. C: Lean and fat mass gain over the 4-week treatment period. D: Metabolic efficiency calculated over the 4-week treatment period. E: iWAT and gWAT weight at the end of the 4-week treatment period. F: BAT and liver weight at the end of the 4-week treatment period. G, H: Plasma levels of glucose, insulin, Gdf15, leptin, adiponectin, FGF21, and lactate at the end of the 4-week treatment period. Statistical analysis was performed using repeated measures two-way ANOVA with Sídák’s multiple comparisons test, and one-way ANOVA with Tukey’s multiple comparisons test. **p < 0.01. *N* = 4-7 per group.

Supplementary Material 2: Supplementary Table 1. Primer sequences used for Real Time Quantitative PCR. Supplementary Table 2. Biochemical assays used for *in vivo* studies.

## Data Availability

All analysed data are displayed. Raw calorimetry data are additionally available on request to the authors. Materials will be made available on reasonable requests to the corresponding author.

## Abbreviations

MSL: Multiple Symmetrical Lipomatosis
EtOH: Ethanol (“alcohol”)
ISR: Integrated stress response
WAT: White adipose tissue
iWAT: Inguinal white adipose tissue
gWAT: Gonadal white adipose tissue
BAT: Brown adipose tissue
DMEM: Dulbecco’s Modified Eagle’s Medium
FBS: Fetal bovine serum
MEF: Mouse embryonic fibroblast
MTT: 3-(4,5-dimethylthiazolyl-2)-2,5-diphenyltetrazolium bromide
EdU: 5-Ethynyl-2-deoxyuridine
PBS: Phosphate-buffered saline
DPBS: Dulbecco’s phosphate-buffered saline
PFA: Paraformaldehyde
PCR: Polymerase chain reaction
DID: Drinking-in-the-dark
EDTA: Ethylenediaminetetraacetic acid
HEPES: N-(2-Hydroxyethyl)piperazine-N’-(2-ethanesulfonic acid)
FCCP: Carbonyl cyanide-p-trifluoromethoxyphenylhydrazone
ANOVA: Analysis of variance
OxPhos: Oxidative phosphorylation

## References

[CR1] Aguer C, et al. Galactose enhances oxidative metabolism and reveals mitochondrial dysfunction in human primary muscle cells. PLoS One. 2011;6(12):e28536. 22194845 10.1371/journal.pone.0028536 PMC3240634

[CR2] Allen GF, et al. Loss of iron triggers PINK1/Parkin-independent mitophagy. EMBO Rep. 2013;14(12):1127–35. 24176932 10.1038/embor.2013.168 PMC3981094

[CR3] Barriocanal-Casado E, et al. Rapamycin administration is not a valid therapeutic strategy for every case of mitochondrial disease. EBioMedicine. 2019;42:511–23. 30898651 10.1016/j.ebiom.2019.03.025 PMC6492073

[CR4] Capel E, et al. MFN2-associated lipomatosis: Clinical spectrum and impact on adipose tissue. J Clin Lipidol. 2018;12(6):1420–35. 30158064 10.1016/j.jacl.2018.07.009

[CR5] Carr AS, et al. MFN2 deletion of exons 7 and 8: founder mutation in the UK population. J Peripher Nerv Syst. 2015;20(2):67–71. 26114802 10.1111/jns.12117

[CR6] Chung KW, et al. Early onset severe and late-onset mild Charcot-Marie-Tooth disease with mitofusin 2 (MFN2) mutations. Brain. 2006;129(Pt 8):2103–18. 16835246 10.1093/brain/awl174

[CR7] Civiletto G, et al. Rapamycin rescues mitochondrial myopathy via coordinated activation of autophagy and lysosomal biogenesis. EMBO Mol Med. 2018;10(11):e8799. 30309855 10.15252/emmm.201708799 PMC6220341

[CR8] Dabir DV, et al. A small molecule inhibitor of redox-regulated protein translocation into mitochondria. Dev Cell. 2013;25(1):81–92. 23597483 10.1016/j.devcel.2013.03.006 PMC3726224

[CR9] Dang X, et al. Mitochondrial Phenotypes in Genetically Diverse Neurodegenerative Diseases and Their Response to Mitofusin Activation. Cells. 2022;11(6):1053. 35326504 10.3390/cells11061053 PMC8947610

[CR10] Detmer SA, Chan DC. Complementation between mouse Mfn1 and Mfn2 protects mitochondrial fusion defects caused by CMT2A disease mutations. J Cell Biol. 2007;176(4):405–14. 17296794 10.1083/jcb.200611080 PMC2063976

[CR11] de Brito OM, Scorrano L. Mitofusin 2 tethers endoplasmic reticulum to mitochondria. Nature. 2008;456(7222):605–10. 19052620 10.1038/nature07534

[CR12] Elouej S, et al. Loss of MTX2 causes mandibuloacral dysplasia and links mitochondrial dysfunction to altered nuclear morphology. Nat Commun. 2020;11(1):4589. 32917887 10.1038/s41467-020-18146-9 PMC7486921

[CR13] Fan F, et al. Rapamycin as a potential treatment for succinate dehydrogenase deficiency. Heliyon. 2019;5(2):e01217. 30805566 10.1016/j.heliyon.2019.e01217 PMC6374580

[CR14] Garg A, et al. Autosomal recessive progeroid syndrome due to homozygosity for a TOMM7 variant. J Clin Invest. 2022;132(23):e156864. 36282599 10.1172/JCI156864 PMC9711873

[CR15] Gautheron J, et al. Loss of thymidine phosphorylase activity disrupts adipocyte differentiation and induces insulin-resistant lipoatrophic diabetes. BMC Med. 2022;20(1):95. 35341481 10.1186/s12916-022-02296-2 PMC8958798

[CR16] Gorman GS, et al. Mitochondrial diseases Nat Rev Dis Primers. 2016;2:16080. 27775730 10.1038/nrdp.2016.80

[CR17] Hoek JB, Cahill A, Pastorino JG. Alcohol and mitochondria: a dysfunctional relationship. Gastroenterology. 2002;122(7):2049–63. 12055609 10.1053/gast.2002.33613 PMC1868435

[CR18] Ignatenko O, et al. Mitochondrial spongiotic brain disease: astrocytic stress and harmful rapamycin and ketosis effect. Life Sci Alliance. 2020;3(9):e202000797. 32737078 10.26508/lsa.202000797 PMC7409372

[CR19] Johnson SC, et al. mTOR inhibition alleviates mitochondrial disease in a mouse model of Leigh syndrome. Science. 2013;342(6165):1524–8. 24231806 10.1126/science.1244360 PMC4055856

[CR20] Khan NA, et al. mTORC1 Regulates Mitochondrial Integrated Stress Response and Mitochondrial Myopathy Progression. Cell Metab. 2017;26(2):419-428 e5. 28768179 10.1016/j.cmet.2017.07.007

[CR21] Kennedy BK, Lamming DW. The Mechanistic Target of Rapamycin: The Grand ConducTOR of Metabolism and Aging. Cell Metab. 2016;23(6):990–1003. 27304501 10.1016/j.cmet.2016.05.009 PMC4910876

[CR22] Larrea D, et al. MFN2 mutations in Charcot-Marie-Tooth disease alter mitochondria-associated ER membrane function but do not impair bioenergetics. Hum Mol Genet. 2019;28(11):1782–800. 30649465 10.1093/hmg/ddz008 PMC6522073

[CR23] Larsson NG, et al. Pathogenetic aspects of the A8344G mutation of mitochondrial DNA associated with MERRF syndrome and multiple symmetric lipomas. Muscle Nerve Suppl. 1995;3:S102-6. 7603509 10.1002/mus.880181421

[CR24] Lemaitre M, et al. Multiple symmetric and multiple familial lipomatosis. Presse Med. 2021;50(3):104077. 34687914 10.1016/j.lpm.2021.104077

[CR25] Lim K, et al. Lipodistrophy: a paradigm for understanding the consequences of “overloading” adipose tissue. Physiol Rev. 2021;101(3):907–93. 33356916 10.1152/physrev.00032.2020

[CR26] Lockhart SM, Saudek V, O’Rahilly S. GDF15: A Hormone Conveying Somatic Distress to the Brain. Endocr Rev. 2020;41(4):bnaa007. 32310257 10.1210/endrev/bnaa007 PMC7299427

[CR27] Mann JP, et al. A mouse model of human mitofusin-2-related lipodystrophy exhibits adipose-specific mitochondrial stress and reduced leptin secretion. Elife. 2023;12:e82283. 36722855 10.7554/eLife.82283 PMC9937658

[CR28] McWilliams TG, et al. mito-QC illuminates mitophagy and mitochondrial architecture in vivo. J Cell Biol. 2016;214(3):333–45. 27458135 10.1083/jcb.201603039 PMC4970326

[CR29] Montava-Garriga L, et al. Semi-automated quantitation of mitophagy in cells and tissues. Mech Ageing Dev. 2020;185:111196. 31843465 10.1016/j.mad.2019.111196 PMC6961211

[CR30] Moullan N, et al. Tetracyclines Disturb Mitochondrial Function across Eukaryotic Models: A Call for Caution in Biomedical Research. Cell Rep. 2015;10(10):1681–91. 25772356 10.1016/j.celrep.2015.02.034 PMC4565776

[CR31] Naon D, et al. Splice variants of mitofusin 2 shape the endoplasmic reticulum and tether it to mitochondria. Science. 2023;380(6651):eadh9351. 37347868 10.1126/science.adh9351

[CR32] Neusch C, et al. Mitofusin 2 gene mutation (R94Q) causing severe early-onset axonal polyneuropathy (CMT2A). Eur J Neurol. 2007;14(5):575–7. 17437620 10.1111/j.1468-1331.2006.01688.x

[CR33] Pareyson D, et al. Mitochondrial dynamics and inherited peripheral nerve diseases. Neurosci Lett. 2015;596:66–77. 25847151 10.1016/j.neulet.2015.04.001

[CR34] Petrovic N, et al. Thermogenically competent nonadrenergic recruitment in brown preadipocytes by a PPARgamma agonist. Am J Physiol Endocrinol Metab. 2008;295(2):E287-96. 18492776 10.1152/ajpendo.00035.2008

[CR35] Quiros PM, et al. Multi-omics analysis identifies ATF4 as a key regulator of the mitochondrial stress response in mammals. J Cell Biol. 2017;216(7):2027–45. 28566324 10.1083/jcb.201702058 PMC5496626

[CR36] Rath M, et al. Binge ethanol consumption-associated behavioral impairments in male mice using a gelatin-based drinking-in-the dark model. Alcohol. 2021;95:25–36. 34029701 10.1016/j.alcohol.2021.05.001 PMC10629591

[CR37] Rhodes JS, et al. Evaluation of a simple model of ethanol drinking to intoxication in C57BL/6J mice. Physiol Behav. 2005;84(1):53–63. 15642607 10.1016/j.physbeh.2004.10.007

[CR38] Rambold AS, Cohen S, Lippincott-Schwartz J. Fatty acid trafficking in starved cells: regulation by lipid droplet lipolysis, autophagy, and mitochondrial fusion dynamics. Dev Cell. 2015;32(6):678–92. 25752962 10.1016/j.devcel.2015.01.029 PMC4375018

[CR39] Rocha N, et al. Human biallelic MFN2 mutations induce mitochondrial dysfunction, upper body adipose hyperplasia, and suppression of leptin expression. Elife. 2017;6:e23813. 28414270 10.7554/eLife.23813 PMC5422073

[CR40] Richter U, et al. Quality control of mitochondrial protein synthesis is required for membrane integrity and cell fitness. J Cell Biol. 2015;211(2):373–89. 26504172 10.1083/jcb.201504062 PMC4621829

[CR41] Sawyer SL, et al. Homozygous mutations in MFN2 cause multiple symmetric lipomatosis associated with neuropathy. Hum Mol Genet. 2015;24(18):5109–14. 26085578 10.1093/hmg/ddv229

[CR42] Siegmund SE, et al. Low-dose rapamycin extends lifespan in a mouse model of mtDNA depletion syndrome. Hum Mol Genet. 2017;26(23):4588–605. 28973153 10.1093/hmg/ddx341 PMC5886265

[CR43] Softic S, et al. Dietary Sugars Alter Hepatic Fatty Acid Oxidation via Transcriptional and Post-translational Modifications of Mitochondrial Proteins. Cell Metab. 2019;30(4):735-753 e4. 31577934 10.1016/j.cmet.2019.09.003 PMC7816129

[CR44] Thiele TE, Navarro M. “Drinking in the dark” (DID) procedures: a model of binge-like ethanol drinking in non-dependent mice. Alcohol. 2014;48(3):235–41. 24275142 10.1016/j.alcohol.2013.08.005 PMC4004717

[CR45] Valente AJ, et al. A simple ImageJ macro tool for analyzing mitochondrial network morphology in mammalian cell culture. Acta Histochem. 2017;119(3):315–26. 28314612 10.1016/j.acthis.2017.03.001

[CR46] Verhoeven K, et al. MFN2 mutation distribution and genotype/phenotype correlation in Charcot-Marie-Tooth type 2. Brain. 2006;129(Pt 8):2093–102. 16714318 10.1093/brain/awl126

[CR47] Zuchner, S., MFN2 Hereditary Motor and Sensory Neuropathy, in GeneReviews (R), M.P. Adam, et al., Editors. 1993: Seattle (WA). 20301684

