# Supplementary material for: Preclinical evaluation of candidate “kill or cure” strategies to treat MFN2-related lipodystrophy
Source: Mol Med. 2025 Aug 4;31:273. doi: 10.1186/s10020-025-01314-2 (PMC12320274; doi:10.1186/s10020-025-01314-2)
Supplement: Supplementary file 1 — Supplementary Material 1: Supplementary Figure 1: Effect of ethanol on wild-type and Mfn2R707W/R707W mouse embryonic fibroblasts. A: Formazan formed in Mfn2WT/WT, Mfn2WT/R707Wand Mfn2R707W/R707WMEFs treated with a range of EtOH concentrationsfor 24 h expressed as a percentage of vehicle treatment. B: EdU positive cells in MEFs as in, expressed as a percentage of live cells. C: Apotracker positive cells in MEFs as in, expressed as a percentage of live cells. D: Gene expression for Pgc1a, Pgc1b, Mfn1, Mfn2, Drp1, Fis1, Ddit3, Trib3, Atf4, Atf5 and Gdf15 in MEFs as in, normalized to reference gene Tbp. Statistical analysis was performed using Two-way ANOVA with Tukey’s multiple comparisons test. *p < 0.05, **p < 0.01. N = 6, Dotted vertical lines indicate legal limits for blood alcohol when driving. Supplementary Figure 2: Validation of the MitoQC Reporter using FCCP. A: Schematic overview of the Mfn2 R707W x mito-QC breeding strategy, primary cell culture protocol, FCCP treatment, and mitochondrial imaging analysis. B,F: Representative images of primary adipocytes isolated from iWATor BATof R707W x mitoQC mice, treated with 20uM FCCP or Veh for 8h. Green: mitochondrial network, red: mitolysosomes, blue: nuclei. C-E, G-I: mitochondrial content, total mitolysosome, and branch length quantifications of the experiment described in B. Statistical analysis was performed using Two-way ANOVA with Tukey’s multiple comparisons test. *p < 0.05, **p < 0.01, ***p < 0.001, ****p < 0.0001. Supplementary Figure 3: Effect of Ethanolon Mfn2R707W/R707Wfemale mice and wild-typelittermates. A-D: 20% EtOH consumption, Body weight, water consumption, and food intake in WT and Hom males during a 3 month“Drinking in the Dark”protocol with 20% ETOH or water control. E-J: Analysis of fat gain, lean mass gain, brown adipose tissue, inguinal, gonadal white adipose tissue, and liver mass in WT and Hom males at the end of the 3 months DID protocol. K-V: Serum levels of glucose, insulin, adiponectin [file 10020_2025_1314_MOESM1_ESM.pdf]

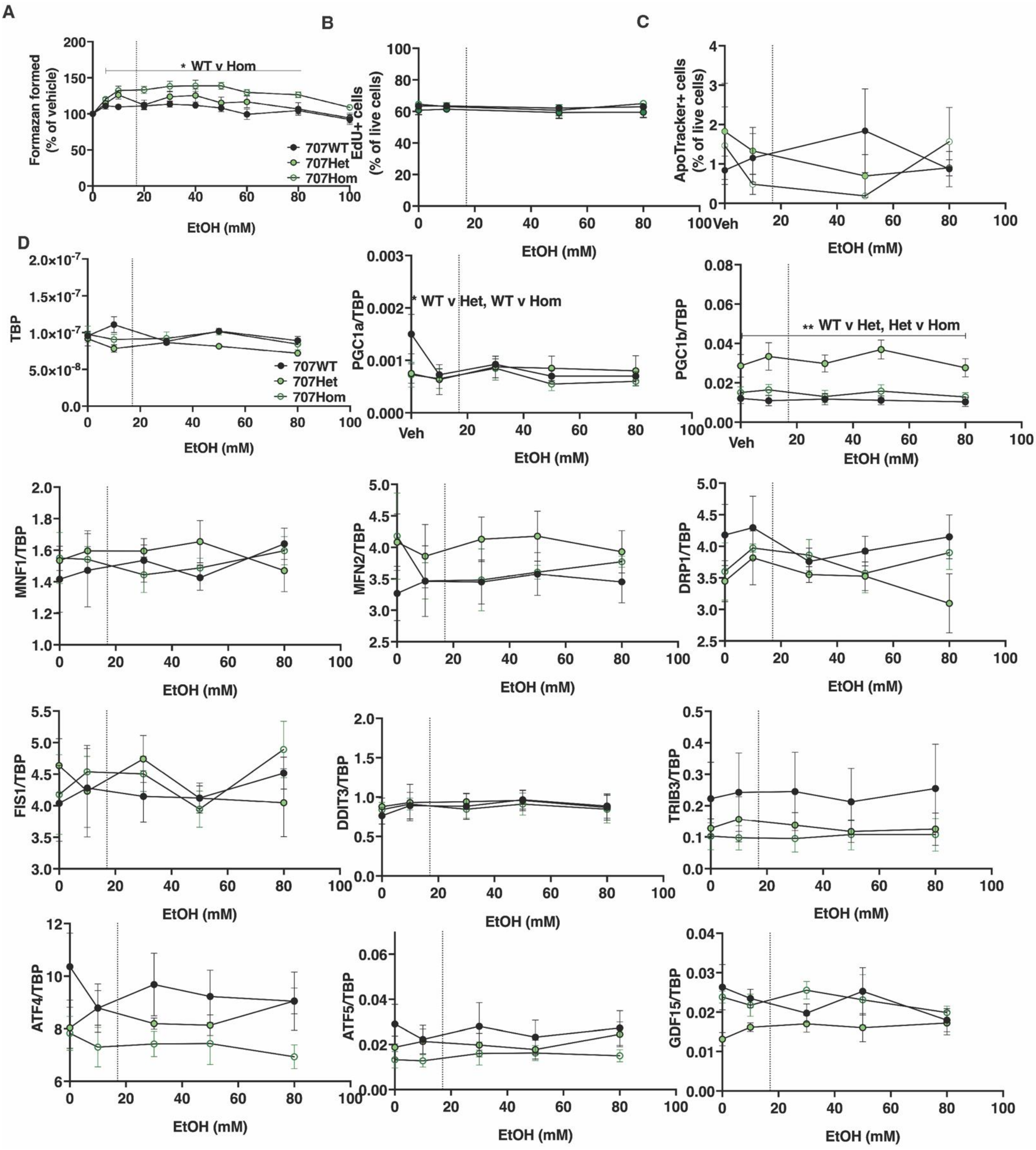

Supplementary Figure 1. Effect of ethanol on on wild-type and *Mfn2*$^{R707W/R707W}$ mouse embryonic fibroblasts

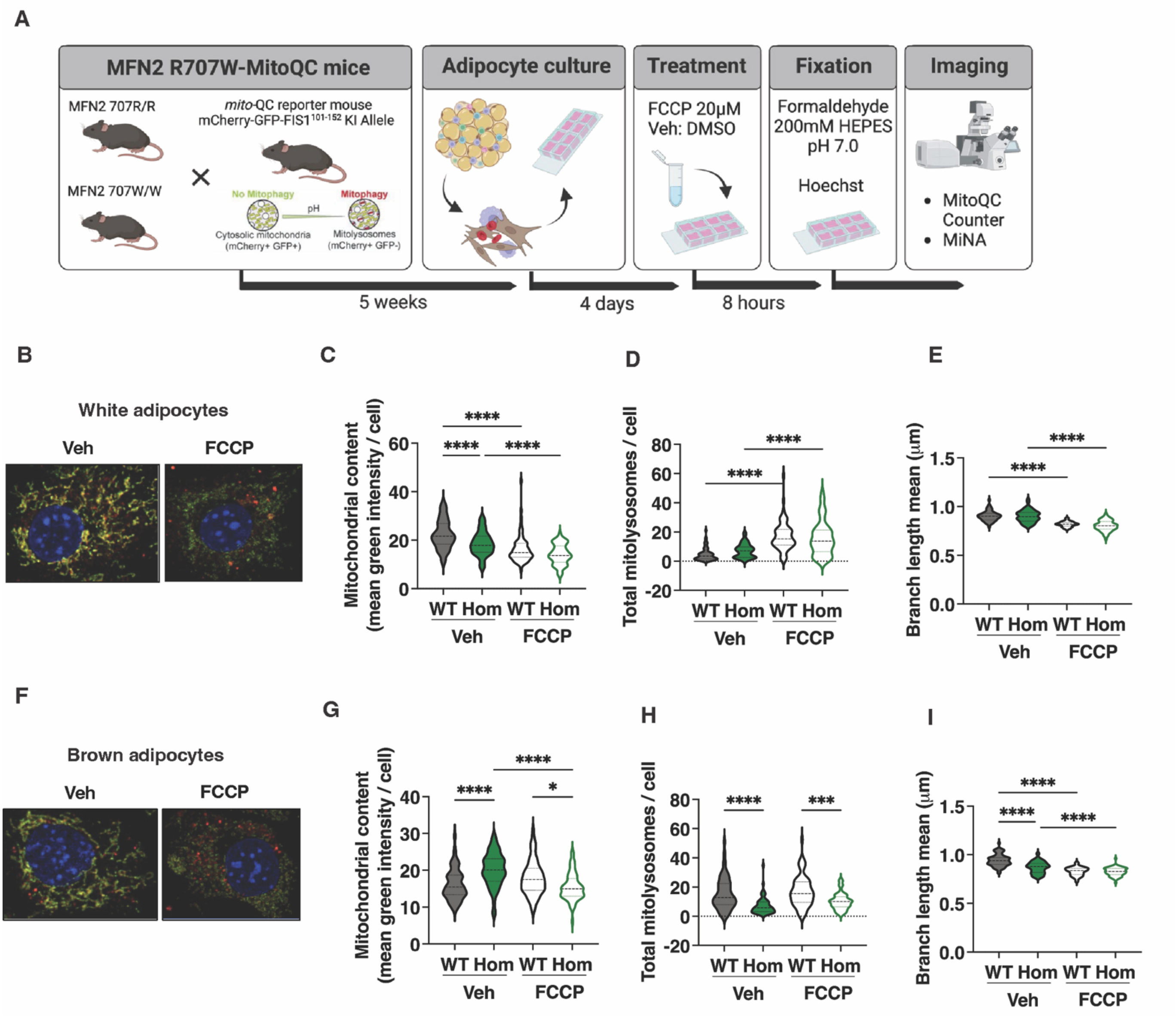

Supplementary Figure 2: Testing of MitoQC Reporter using FCCP.

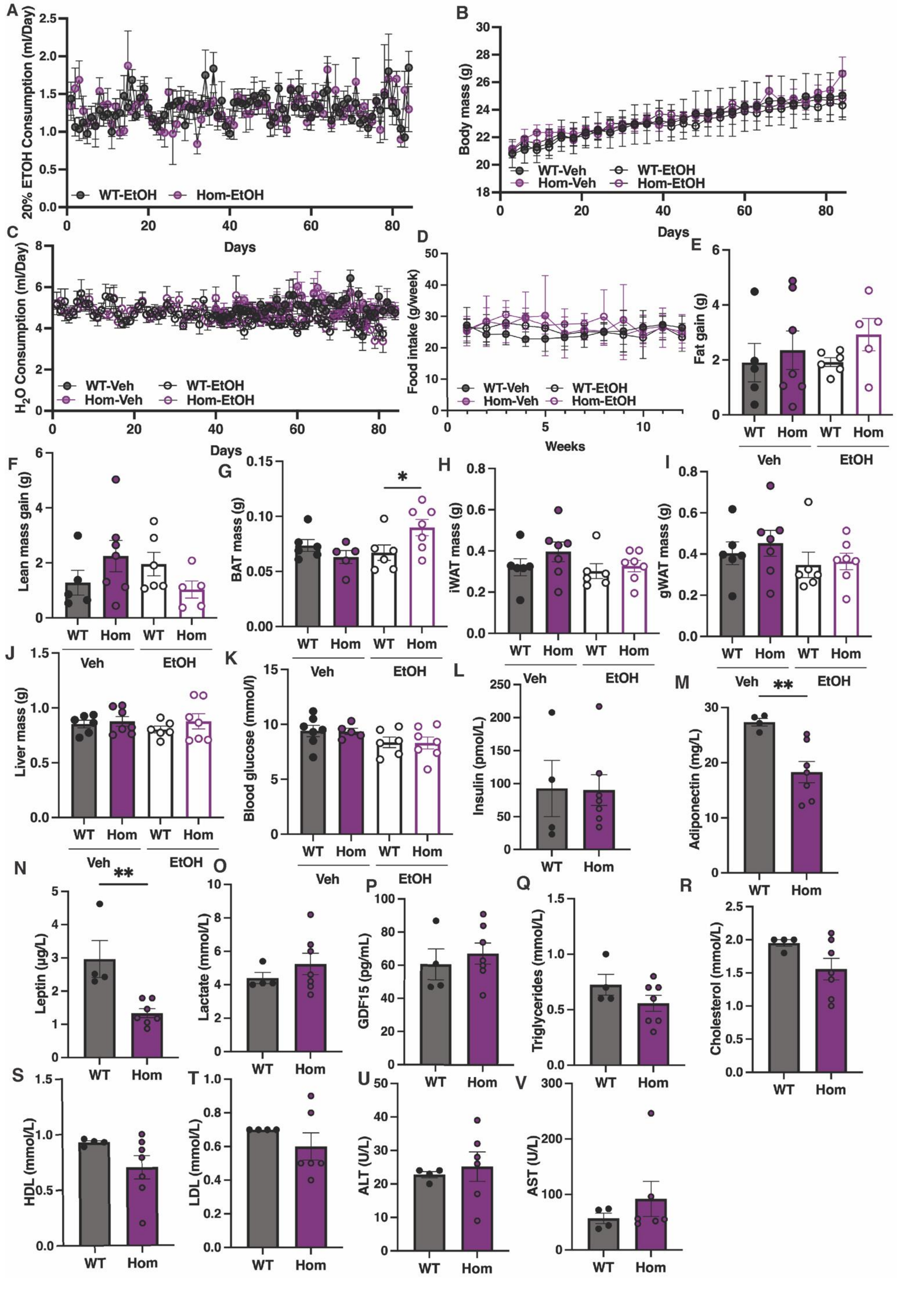

Supplementary Figure 3. Effect of ethanol on female wild-type and *Mfn2*$^{R707W/R707W}$ mice

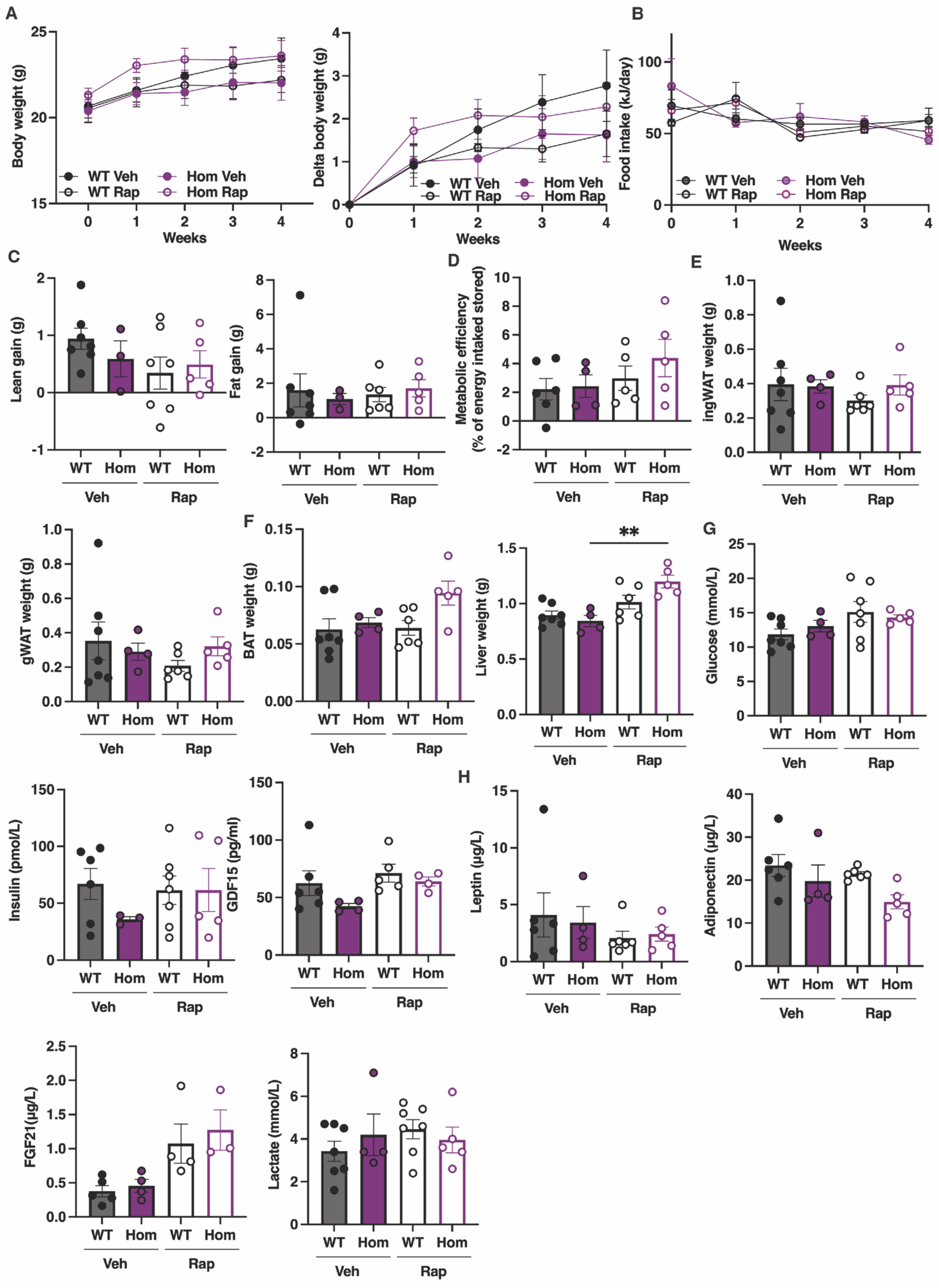

Supplementary Figure 4. Effect of rapamycin on female wild-type and *Mfn2*$^{R707W/R707W}$ mice
