# Supplementary material for: Preclinical evaluation of candidate “kill or cure” strategies to treat MFN2-related lipodystrophy
Source: Mol Med. 2025 Aug 4;31:273. doi: 10.1186/s10020-025-01314-2 (PMC12320274; doi:10.1186/s10020-025-01314-2)
Supplement: Supplementary file 2 — Supplementary Material 2: Supplementary Table 1. Primer sequences used for Real Time Quantitative PCR. Supplementary Table 2. Biochemical assays used for in vivo studies. [file 10020_2025_1314_MOESM2_ESM.docx]

**Supplementary Tables**

**Supplementary Table 1. Primer sequences used for Real Time Quantitative PCR**

| **Gene** | **Forward primer** | **Reverse primer** |
| --- | --- | --- |
| *Tbp* | ACGGACAACTGCGTTGATTT | TTCTTGCTGCTAGTCTGGATTG |
| *Atf4* | CAACCCCCACCGGCCTAAG | GTTGTGGGGCTTTGCTGGATT |
| *Atf5* | ATGAGGTCCTTGGGGGTGCC | AAGTCCACCCGCTCAGTCAT |
| *Ddit3* | TCCTGTCCTCAGATGAAATTGG | GCAGGGTCAAGAGTAGTGAAG |
| *Drp1* | GGGCACTTAAATTGGGCTCC | TGTATTCTGTTGGCGTGGAAC |
| *Fis1* | CAAAGAGGAACAGCGGGACT | ACAGCCCTCGCACATACTTT |
| *Gdf15* | AGTGTCCCCACCTGTATCG | TGTCCTGTGCATAAGAACCA |
| *Mfn1* | ATGGCAGAAACGGTATCTCCA | GCCCTCAGTAACAAACTCCAGT |
| *Mfn2* | TGCACCGCCATATAGAGGAAG | TCTGCAGTGAACTGGCAATG |
| *Pgc1a* | GAAAGGGCCAAACAGAGAGA | GTAAATCACACGGCGCTCTT |
| *Pgc1b* | GTCCCTGACGGTGGAGCTTT | GGTGTCTGGCTTGAAGGGGT |
| *Trib3* | GCAGATGGCTAGTGCCGTGG | CATCACGCAGGCATCTTCCAG |

**Supplementary Table 2. Biochemical assays used for *in vivo* studies**

| **Analyte** | **Reagent manufacturer** | **Product code** |
| --- | --- | --- |
| Insulin and leptin | MesoScale Discovery (Rockville, MD, USA) | K15124C-3 |
| Lactate | Siemens Healthcare | DF16 |
| Triglycerides | Siemens Healthcare | DF69A |
| Total cholesterol | Siemens Healthcare | DF27 |
| HDL cholesterol | Randox | CH2849 |
| LDL cholesterol | Randox | CH2656 |
| Adiponectin | MesoScale Discovery | K152BYC-2 |
| Gdf15 | R&D Systems | DuoSet ELISA (modified to run as an electrochemiluminescence assay on the Meso Scale Discovery platform) |
| Fgf21 | R&D Systems | Quantakine ELISA |
| AST | Siemens Healthcare | DF41A |
| ALT | Siemens Healthcare | DF143 |
